# Ultrasonic rolling of 42CrMo Steel: Simulation-Based process optimization for high-efficiency and sustainable manufacturing^☆^

**DOI:** 10.1016/j.ultsonch.2025.107565

**Published:** 2025-09-11

**Authors:** Haojie Wang, Xiaoqiang Wang, Eric Velázquez-Corral, Ramón Jerez-Mesa

**Affiliations:** a School of Mechatronics Engineering, Henan University of Science and Technology, Luoyang 471003, China; b Department of Mechanical Engineering, Universitat Politècnica de Catalunya, Escola d’Enginyeria de Barcelona Est, Av. Eduard Maristany, 16, 08019 Barcelona, Spain

**Keywords:** Ultrasonics, Green manufacturing, Simulation, Efficient, Performance optimization

## Abstract

Ultrasonic rolling has emerged as a highly promising surface strengthening technique due to its ability to enhance surface properties under low energy consumption conditions. In this study, a thermo-mechanically coupled simulation model was developed to investigate the ultrasonic rolling process of 42CrMo steel, aiming to achieve a balance between high surface performance and low carbon emissions through process optimization. An orthogonal array of 25 simulation experiments were designed and conducted to analyze the effects of process parameters—including workpiece rotational speed, static pressure, feed rate, and ultrasonic amplitude—on surface properties and thermal behavior. Based on the simulation data, a response surface methodology (RSM) was employed to establish predictive models for surface performance. To address the conflicting objectives among various surface performance indicators and significantly improve both surface quality and processing efficiency, a novel hybrid optimization algorithm, the Particle Swarm Simulated Annealing Optimization (PSSAO), was proposed. Multi-objective optimization was performed to determine an optimal parameter domain that enables effective strengthening in a single-pass rolling process. The optimal processing window obtained from PSSAO was experimentally validated, and the results confirmed the reliability of the simulation model and the feasibility of this sustainable manufacturing strategy. The findings demonstrate that ultrasonic rolling of 42CrMo steel, when optimized through thermo-mechanical simulations and advanced parameter design, can effectively enhance surface properties, reduce energy consumption, shorten processing time, and lower carbon emissions—providing a viable pathway toward sustainable manufacturing. The PSSAO algorithm identified the optimal processing parameter ranges as a feed rate of 0.05–0.07 mm/r, static pressure of 520–700 N, ultrasonic amplitude of 7–9 μm, and spindle speed of 50–180 r/min. Under these conditions, the maximum residual compressive stress reaches −1283 to −1296 MPa, average surface roughness (Ra) is reduced to 0.120–0.156 μm, and surface hardness increases to 60.9–61.6 HRC.

## 1 Introduction

42CrMo steel, known for its outstanding wear resistance and toughness, has been extensively applied in critical components across various manufacturing sectors such as aerospace, rail transit, construction machinery, and wind power generation [[1], [2], [3]]. It is particularly prevalent in load-bearing components such as drive shafts, bearings, and gears. Despite its widespread application as a high-strength steel, 42CrMo is still susceptible to failure. Under heavy load conditions, the initiation of cracks or pitting during service can lead to surface degradation of components, drastically shortening service life and even causing severe accidents involving equipment failure or personal injury. To address this issue and further enhance the surface properties of 42CrMo steel, it is imperative to adopt surface strengthening methods that significantly improve mechanical performance and reduce failure risks. Traditionally, parts are subjected to grinding operations following turning to improve surface quality. While grinding can enhance surface finish and introduce a certain depth of residual compressive stress layer [4,5], it requires large quantities of grinding fluid, which is typically non-reusable and harmful to the environment [6]. Moreover, switching from a lathe to a grinding machine between operations reduces overall production efficiency. Grinding also risks surface burns, potentially diminishing surface properties—factors that conflict with the principles of environmentally friendly and green manufacturing [7]. As green manufacturing technologies evolve, the concept of “rolling instead of grinding” has gained popularity. Ultrasonic rolling composite processing (URCP), a novel technique that superimposes ultrasonic vibrations onto conventional rolling (CR), induces severe plastic deformation by promoting surface material flow through the ultrasonic effect [[8], [9], [10]]. During this process, cooling lubricants reduce surface temperature and are recyclable within the system, adhering to sustainable manufacturing standards. Furthermore, ultrasonic systems typically operate at a few hundred W—far lower than the energy consumption of grinding—demonstrating notable advantages in energy efficiency and environmental protection. Compared with other surface strengthening techniques such as laser treatment, electrochemical machining, or ultrasonic shot peening [[11], [12], [13]], URCP offers additional benefits including eco-friendliness, simple equipment structure, and portability—aligning well with global sustainable development goals.

For instance, Zha et al. [14] studied the kinetic energy and hardness evolution of TC4 titanium alloy under single-impact ultrasonic rolling, finding increased kinetic energy and a hardness peak beneath the surface as ultrasonic amplitude rose. Liu et al. [15] showed that ultrasonic rolling created a 600 μm-thick work-hardened layer in 3600 aluminum alloy, increasing fatigue life fivefold under 100 MPa stress. Liu et al. [16], using ABAQUS, found that although residual compressive stress depth increased with more rolling passes, the peak stress occurred after a single pass. Zhou et al. [17] enhanced the mechanical properties of additively manufactured Ti6Al4V through URCP, improving ductility, elongation, and hardness by twofold. Li et al. [18] applied URCP to improve the wear resistance of Ti6Al4V, observing increased surface hardness, a deepened hardened layer, and reduced friction coefficient and wear rate. Li et al. [19] used Bayesian-optimized neural networks to determine optimal URCP parameters for titanium alloy blades, achieving maximum residual stress and minimum surface roughness when the rolling depth was 0.15 mm and feed rate was 100 mm/min. Further studies include Liu et al. [20] demonstrating refined grain size (from 100 μm to ∼50 μm) and enhanced hardness (350 HV) in high-entropy alloy coatings, with wear rate reduced by 90 %. Ren et al. [21] showed that URCP improved the interfacial adhesion strength of epoxy coatings on P110 steel by 30 %, attributed to improved surface wettability. Liu et al. [22] found that URCP raised residual stress in 7075 aluminum to 600 MPa and hardness to 170 HV, boosting fatigue life by over 100 times.

Gao et al. [23] reported significant fatigue life improvement of TC4 welds after URCP, due to residual stress suppressing crack propagation. Shi et al. [24] developed a surface roughness prediction model for gear teeth treated with URCP, finding roughness Sa reduced to one-third and Sq to 0.2 μm, confirming substantial surface quality enhancement. Li et al. [25] simulated bubble pressure changes due to cavitation under ultrasonic rolling, noting an increase from 5000 MPa to nearly 40,000 MPa with rising amplitude—contributing to improved surface properties. Li et al. [26] observed that URCP reduced the friction coefficient of laser-melted Li alloys from 0.4 to 0.3 due to grain refinement and increased dislocation density. Tong et al. [27] addressed low URCP efficiency on flat plates by developing an eccentric-plane device, achieving surface hardness of 500 HV and residual stress beyond −640 MPa for TC4 alloy. Wang et al. [28] improved corrosion resistance of carburized gear steel, finding optimal performance at 0.6 MPa static pressure. Qin et al. [29] noted that excessive URCP passes could introduce surface cracks, negatively impacting fatigue resistance. Ji et al. [30] found that URCP increased EA4T axle steel’s fatigue strength by 100 MPa in dry air and 230 MPa in humid conditions, showing even greater effectiveness in corrosive environments. From the above studies, it is evident that URCP has primarily been applied to softer materials like titanium and aluminum alloys. Research on its effects on hardened high-strength steels such as quenched 42CrMo is rare, despite the latter's widespread industrial applications. As the demands for longer service life, higher fatigue resistance, and greener manufacturing grow, it becomes increasingly crucial to improve the surface performance of high-strength steels like 42CrMo in a sustainable manner.

Moreover, most existing studies focus on the effects of multiple rolling passes, which, like traditional grinding, reduce processing efficiency and increase energy consumption and carbon emissions. Therefore, identifying an optimal set of URCP parameters capable of significantly enhancing surface properties in a single pass is essential. With the rise of machine learning, it is now feasible to quickly determine optimal processing domains tailored to 42CrMo steel using intelligent algorithms—an approach aligned with the goals of green, high-efficiency manufacturing. Previous URCP studies often overlooked the coupling between thermal and mechanical fields. Our earlier work [31] confirmed that URCP induces adiabatic heating, which softens the material and promotes plastic deformation. However, precisely establishing a thermo-mechanical coupling simulation model for URCP and efficiently identifying optimal process parameters remain critical challenges. Additionally, improving surface performance through single-pass URCP to enhance processing efficiency and reduce environmental impact is another pressing need. However, existing studies have not systematically elucidated the thermo-mechanical coupling effects in URCP, nor have they provided efficient parameter optimization strategies to guide single-pass processing. This research gap directly motivates the present work.

Thus, this study combines theoretical analysis, simulation modeling, experimental processing, and multi-objective optimization to comprehensively investigate surface performance enhancement of 42CrMo steel. A thermo-mechanically coupled finite element model is developed and validated through extensive URCP simulations under various parameter combinations. Based on this, a novel optimization strategy integrating swarm intelligence and deep learning is proposed to identify optimal URCP parameter domains. Accurate prediction and optimization of process parameters enable high-quality surface enhancement in a single pass, achieving efficient URCP with minimal energy consumption. This work provides a new perspective for realizing sustainable manufacturing and carbon emission reduction. The novelty of this research lies in establishing a validated thermo-mechanical coupling model for URCP that captures the role of adiabatic heating, introducing an intelligent optimization framework that combines swarm intelligence with deep learning to efficiently explore process domains, and demonstrating the feasibility of single-pass URCP as an effective pathway to achieve high-quality surface properties while reducing energy consumption and emissions for the first time.

## 2 URCP simulation modeling

### 2.1 Principle of ultrasonic rolling surface strengthening

**Fig. 1:**
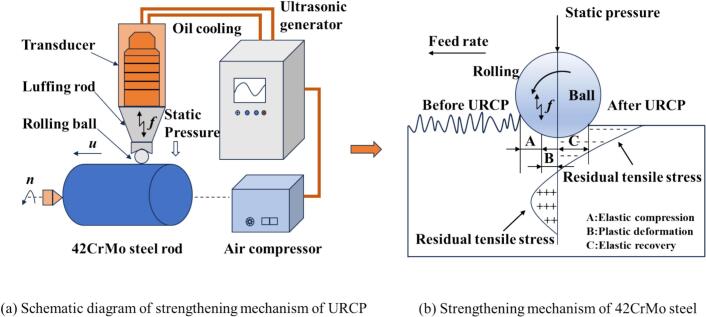
Schematic diagram of the URCP[32].

As a representative green manufacturing technique with low energy consumption and minimal environmental impact, URCP involves applying high-frequency ultrasonic vibrations to a rolling ball at the end of the actuator, which then plastically deforms the workpiece surface through contact, as illustrated in Fig. 1a. The ultrasonic rolling device consists of a rolling ball, a horn (amplitude transformer), a transducer, and an ultrasonic generator. The system is also connected to a cooling fluid delivery mechanism and an air compressor to supply static pressure and ensure surface lubrication and cooling at the tool end. First, the ultrasonic generator produces a high-frequency alternating electrical signal. Then, the transducer converts this signal into mechanical vibrations by utilizing the piezoelectric ceramics within, which undergo periodic expansion and contraction under alternating current—effectively transforming 220 V AC power into vibrational energy. Next, the stepped horn amplifies these mechanical vibrations, transforming low-amplitude input into high-frequency mechanical oscillations. The horn used in this study is designed to operate at a resonant frequency of 28 kHz. The horn structure must match the acoustic impedance and energy transmission characteristics of ultrasonic waves in solid materials, achieving amplitude magnification by several tens to even hundreds of times. The stepped horn ensures efficient wave propagation with minimal energy loss and reflection, enabling effective ultrasonic rolling. Although the amplitude can be finely adjusted by varying the input voltage to the transducer, large-range control is not possible due to the fixed structural design and amplification ratio of the horn. Altering the ultrasonic frequency without matching the system’s natural resonance would lead to impedance mismatch and inefficient wave transmission, or even complete failure of vibration output. Therefore, the horn and the transducer must form a matched pair, with each horn structure corresponding to a specific resonant frequency to achieve stable and amplified ultrasonic vibration through resonance. Amplitude adjustments are limited to small variations controlled via the input voltage. To operate at different ultrasonic frequencies, new horns with different structural parameters would be needed. Hence, this study focuses exclusively on a 28 kHz stepped horn for both simulation and experimental validation; other frequencies are not considered here. The relationship between acoustic impedance, wave velocity, and density during the URCP process is given in Eq. (1):

(1) Z=ρ·c

Where ρ is the density of the horn material, c is the propagation velocity of ultrasonic waves in the horn.

Based on the above analysis, when the ultrasonic resonance frequency is fixed at 28 kHz, energy generated by the transducer is efficiently transmitted to the horn tip without reflection, allowing rapid and effective energy transfer for surface strengthening of metallic materials. The entire URCP system is mounted on a CNC lathe, where the workpiece rotates continuously, and the actuator moves along the feed direction to perform URCP. Simultaneously, the rolling ball vibrates vertically at high ultrasonic frequencies, achieving rapid contact–separation cycles. This results in uniform plastic deformation on the surface, enhancing the mechanical properties of the material.

Fig. 1b illustrates the working principle of ultrasonic surface strengthening. It explains how the surface of 42CrMo steel is improved under high-frequency impacts from the rolling ball, leading to superior surface quality and the generation of beneficial residual compressive stress. Initially, the material surface is rough and uneven. Under the compressive force of the rolling ball, these surface defects are smoothed out. In region A, where contact begins, the material undergoes elastic deformation. In region B, where the ball makes the deepest contact, plastic deformation occurs as the stress exceeds the yield strength of 42CrMo steel, resulting in permanent, non-recoverable deformation. In region C, which is the exit region, the material enters elastic recovery. However, due to work hardening, surface deformation resists full elastic recovery, thereby generating residual compressive stress on the surface.

To balance internal stress, the deeper subsurface layers maintain a residual tensile stress state. Throughout the process, the URCP treatment causes the metal to flow in the direction of the rolling motion, smoothing out machining marks and defects from prior processes—a phenomenon known as the “peak shaving and valley filling” effect. This results in a uniform work-hardened layer, significantly reducing surface roughness and improving surface quality. At the same time, the surface hardness and residual compressive stress are markedly enhanced. This constitutes the fundamental mechanism and performance-enhancing effect of the ultrasonic rolling surface strengthening system. Ultrasonic vibration enhances the strengthening effect not merely by superimposing dynamic stress on the quasi-static load or by inducing localized heating, but by intrinsically altering the material’s deformation behavior. It promotes dislocation mobility and facilitates dipole annihilation, thereby accelerating subgrain formation during plastic deformation. This acoustoplastic effect leads to a more pronounced microstructural refinement and contributes significantly to the observed increase in residual compressive stress, surface hardness, and reduction in surface roughness during the URCP [33].

### 2.2 URCP motion trajectory

**Fig. 2:**
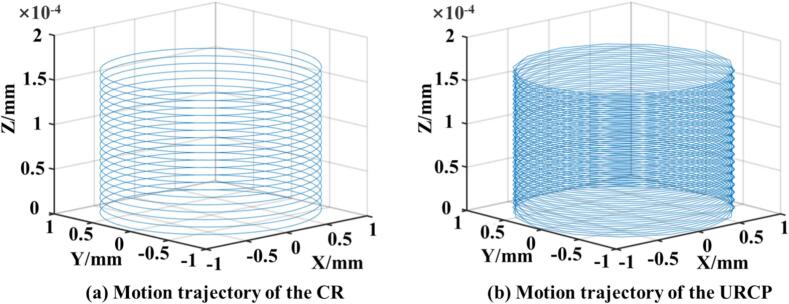
Motion of the rolling ball in 3D space before and after ultrasonic vibration is applied.

To implement the motion trajectory of the rolling ball during the URCP in ABAQUS, and to further develop a kinematic model for the rolling ball under ultrasonic conditions, it is first necessary to clarify the motion behavior of the rolling ball within the ultrasonic actuator. This analysis lays the kinematic foundation for subsequent URCP simulations and process parameter optimization. As illustrated in Fig. 2, MATLAB software was used to plot the three-dimensional motion trajectories of the rolling ball during the rolling process, both with and without ultrasonic excitation. Fig. 2a shows the motion state of the rolling ball without ultrasonic excitation, as same as CR. The corresponding kinematic equation is given in Eq. (2). It can be observed that in conventional rolling process, the trajectory of the ball is a continuously extended 3D helix along the feed direction of the lathe. In contrast, Fig. 2b depicts the motion state of the ball under ultrasonic excitation, with the kinematic equation provided in Eq. (3). In this case, the ball exhibits high-frequency vibration in the X and Y directions while extending helically along the Z direction. The ultrasonic frequency is set at 28 kHz, with an amplitude of 8 μm. The feed rate is 0.1 mm/r, and the workpiece rotates at 50 r/min.

(2) $$\left\{\begin{array}{c} x=R\times \cos\left(\mathrm{wt}\right)\\ y=R\times \sin\left(\mathrm{wt}\right)\\ z=\frac{\mathrm{ft}}{60} \end{array}\right)$$

(3) $$\left\{\begin{array}{c} x=R\times \cos\left(\mathrm{wt}\right)+A\times \sin\left(2\pi f_{u}t\right)\\ y=R\times \sin\left(\mathrm{wt}\right)+A\times \sin\left(2\pi f_{u}t\right)\\ z=\frac{\mathrm{ft}}{60} \end{array}\right)$$

**Fig. 3:**
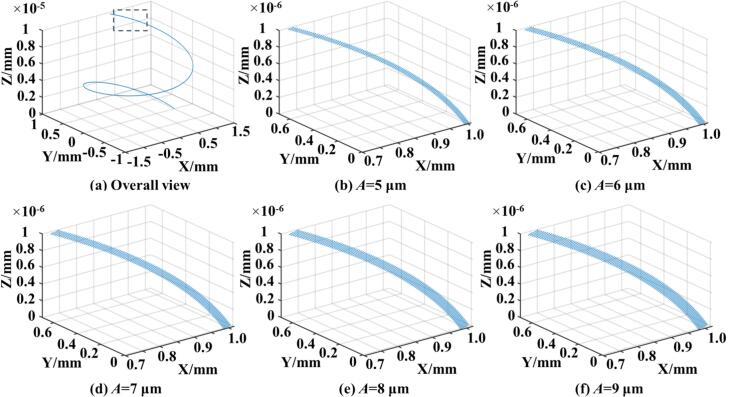
3D motion trajectories of the rolling ball under different vibration amplitudes.

**Fig. 4:**
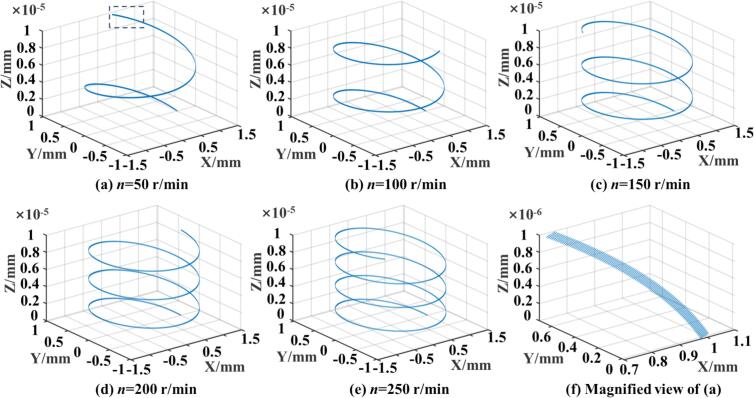
3D motion trajectories of the rolling ball under different spindle speeds.

To further explore the influence of different process parameters on the motion trajectory, the motion states under various ultrasonic amplitudes were plotted, as shown in Fig. 3. As the ultrasonic amplitude increases, the vibration amplitude of the rolling ball also increases within a unit of time, resulting in more intense plastic deformation [34]. If greater ultrasonic strengthening energy and enhanced material deformation are desired, larger ultrasonic amplitudes should be selected. Fig. 3 shows the spatial trajectories of the rolling ball under different amplitudes, confirming that increased amplitude leads to increased vibrational magnitude. Fig. 4 illustrates the rolling ball's motion under varying workpiece rotational speeds. With increasing speed, the number of revolutions completed by the rolling ball per unit time also increases. These two variables—ultrasonic amplitude and workpiece speed—are key processing parameters for both simulation and experimental studies. They play a crucial role in achieving the desired surface properties and surface quality of 42CrMo steel. Different combinations of amplitudes and speeds lead to varying degrees of uniform or non-uniform plastic deformation.

It is important to note that variations in static pressure only affect the distribution of contact stress and the extent of deformation, as they are applied in the normal direction and do not influence the motion trajectory. Thus, motion diagrams under varying static pressures are not presented. Similarly, the feed speed changes linearly and is not considered in this section. In summary, to identify the optimal parameter combinations for improved surface quality and superior surface properties of 42CrMo steel, subsequent simulations and experiments are conducted within the following ranges: workpiece speed from 50 r/min to 250 r/min, ultrasonic amplitude from 5 μm to 9 μm, and feed rate from 0.05 mm/r to 0.25 mm/r. The ultrasonic frequency remains fixed at 28 kHz, determined by the structure of the horn and transducer. This analysis also provides a theoretical and kinematic basis for applying ultrasonic excitation and motion paths of the rolling ball during the URCP simulation process.

### 2.3 Finite element modeling process of URCP

The material used for the URCP simulation is 42CrMo steel bar stock, and the Johnson-Cook (JC) material model is adopted to define the constitutive behavior, as expressed in Eq. (4).

(4) $$\mathrm{JC}={\left(1-\frac{Y-Y_{r}}{Y_{m}-Y_{r}}\right)}^{m}\left(A_{c}+B\eta^{q}\right)\left(1+\alpha \ln\frac{\eta_{1}}{\eta_{2}}\right)$$

This JC model accounts for the adiabatic temperature rise induced during URCP, enabling a coupled stress–temperature analysis to more accurately represent the surface property outcomes. In the equation, Y represents the real-time temperature of the 42CrMo steel, $Y_{r}$ is the ambient room temperature, and $Y_{m}$ is the melting temperature of 42CrMo steel. m denotes the thermal softening coefficient, $A_{c}$ is the initial yield stress before URCP, B is the strain hardening coefficient, η is the equivalent plastic strain, q is the strain hardening exponent, α is the strain rate sensitivity coefficient, $\eta_{1}$ is the current strain rate, and $\eta_{2}$ is the reference strain rate. The mechanical and thermal properties are listed in Table 1, and the specific parameters of the JC model are given in Table 2.

**Table 1:** Mechanical and thermal properties of 42CrMo steel [35].

Parameter	Value
Density	7850 kg/m^3^
Elastic modulus	212 GPa
Poisson’s ratio	0.28
Specific heat capacity	470 J/(kg·K)
Thermal expansion coefficient	11 × 10^−6^/K
Inelastic heat fraction	0.9

**Table 2:** Constitutive model equations for 42CrMo steel.

Parameter	Value
$A_{c}$	1059 MPa
B	528 MPa
α	0.008
m	1
q	0.211
Initial Temperature ($Y_{r}$)	20 °C
Melting Temperature ($Y_{m}$)	1394 °C

**Fig. 5:**
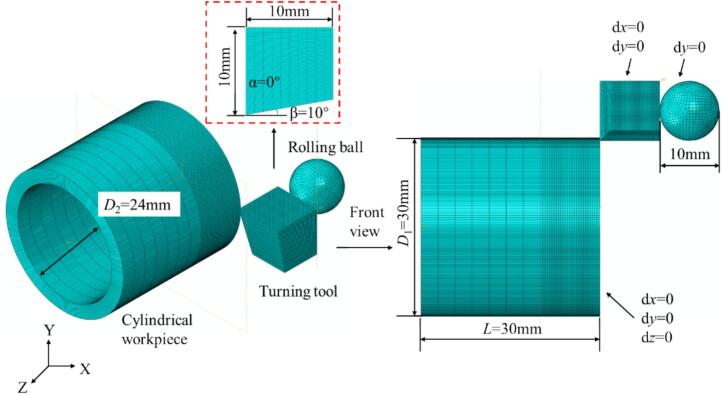
Finite element modeling of URCP on 42CrMo steel.

The thermomechanically coupled URCP simulation model was developed using ABAQUS. Considering that URCP is a high strain-rate plastic deformation process characterized by intense plastic flow, the Explicit module is employed for the analysis steps. The developed URCP simulation model is shown in Fig. 5. The workpiece is a 42CrMo steel hollow cylinder that has undergone a prior turning process. The turning tool has a rake angle of 0° and a clearance angle of 10°. The surface damage from the turning process is also modeled using the Johnson-Cook damage model, with parameters selected as 0.0368, 2.34, –1.484, 0.0035, and 0.411. The turning conditions are: cutting speed of 180 m/min, depth of cut of 0.2 mm, and feed rate of 0.05 mm/r. After turning, the ultrasonic rolling simulation is performed.

Since the rolling ball is made of tungsten carbide with a surface hardness significantly higher than that of 42CrMo steel, it is modeled as an analytical rigid body to reduce computation time. In ABAQUS, analytical rigid bodies do not require meshing. The 42CrMo steel part, however, is fully meshed to avoid mesh distortion and hourglass defects that could affect simulation accuracy. C3D8RT thermomechanical coupled elements are used. Mesh refinement is applied to the surface layer of the workpiece to a depth of 1 mm with an element size of 0.1 mm. Additional refinement is performed at the starting section 10 mm along the rolling direction, with 0.3 mm refinement in the radial direction. The rest of the global mesh is approximately 3 mm, totaling 125,600 elements. To further improve computational efficiency, the simulated workpiece is modeled as a hollow cylinder with an outer diameter of 30 mm, an inner diameter of 24 mm, a thickness of 3 mm, and a length of 300 mm. Surface-to-surface contact is defined, with the tangential behavior set to penalty friction and a friction coefficient of 0.2. Normal contact is defined as hard contact. The friction coefficient is set to 0.2 to reflect the mildly lubricated contact condition typically present in URCP, where lubricants are often applied to reduce friction, heat generation, and tool wear. This value is also consistent with those adopted in previous finite element studies of surface strengthening, ensuring both physical realism and numerical stability [36]. Given the thermomechanical coupling, heat transfer is enabled in the contact properties, with a gap range of 0–0.3 and a heat transfer coefficient between 0 and 1000. To compare the effects of URCP and CR on the surface properties of 42CrMo steel, ultrasonic excitation is defined in ABAQUS using the amplitude module. The excitation force in URCP is defined as shown in Eq. (5),

(5) $$F\left(t\right)=A_{i}+\sum {\;}_{n=1}^{\infty }\left(a_{n}\cos\omega t+b_{n}\sin\omega t\right)$$

where $A_{i}$ represents the initial static pressure, $a_{n},b_{n}$ denotes the amplitude of different ultrasonic modes, ω is the angular frequency (derived from the fixed ultrasonic frequency of 28 kHz), and t is the duration of ultrasonic excitation. The output is a sinusoidal wave when $n=1,a_{n}=0$ For the CR, no ultrasonic amplitude is applied, and only the static pressure parameter is defined, without invoking the amplitude module.

An orthogonal experimental design consisting of 25 groups and four factors at five levels is developed, as shown in Table 3. This design enables a comprehensive assessment of the coupled effects of multiple process parameters on the surface performance outcomes during ultrasonic rolling.

**Table 3:** Orthogonal design of URCP simulation parameters for 42CrMo steel.

Groups	Rotational speed: *n* (r/min)	Amplitude: *A* (μm)	Static pressure: *F* (N)	Feed rate: *u* (mm/r)
1	50	5	300	0.05
2	50	6	400	0.10
3	50	7	500	0.15
4	50	8	600	0.20
5	50	9	700	0.25
6	100	5	400	0.15
7	100	6	500	0.20
8	100	7	600	0.25
9	100	8	700	0.05
10	100	9	300	0.10
11	150	5	500	0.25
12	150	6	600	0.05
13	150	7	700	0.10
14	150	8	300	0.15
15	150	9	400	0.20
16	200	5	600	0.10
17	200	6	700	0.15
18	200	7	300	0.20
19	200	8	400	0.25
20	200	9	500	0.05
21	250	5	700	0.20
22	250	6	300	0.25
23	250	7	400	0.05
24	250	8	500	0.10
25	250	9	600	0.15

### 2.4 Simulation results of URCP

In general, the evaluation of the surface properties of materials after ultrasonic rolling is primarily based on surface residual stress, hardness, and surface roughness. Among these, the residual compressive stress can be inferred from the minimum principal stress in the von-Mises stress contour plot after ultrasonic rolling, which typically reflects changes in surface residual stress under the Johnson-Cook mechanical model during the URCP.

Hardness, however, cannot be directly extracted in ABAQUS post-processing. Instead, it must be calculated indirectly by converting the equivalent plastic strain into hardness using a strain-hardening curve. Similarly, surface roughness cannot be directly computed from the simulation. It is usually represented by analyzing the displacement variations of the surface nodes in the finite element mesh of the 42CrMo steel model. Specifically, the surface roughness is estimated by calculating the average absolute deviation of the displacement of seven consecutive nodes along the URCP path, in order to minimize measurement error, as shown in Eq. (6) [37]:

(6) $$\mathrm{SR}=\frac{1}{n}\sum_{i=1}^{n}\left|\gamma_{i}\right|$$

Likewise, the surface hardness after URCP cannot be directly obtained from ABAQUS. It must be derived by converting the equivalent plastic strain (PEEQ) from the post-processing results into Rockwell hardness. The relationship between the Rockwell hardness and equivalent plastic strain after URCP is expressed in Eq. (7) [38]:

(7) $$\mathrm{HRC}=H_{0}+kP^{n}$$

In this equation, P represents the equivalent plastic strain on the material surface after URCP. $H_{0}$ is the initial Rockwell hardness of 42CrMo steel, taken as 50 HRC. k is an empirical constant, typically set to 20. n is the strain-hardening exponent in the constitutive model of 42CrMo steel, which is set to 0.5 based on the results of mechanical property testing. Table 4 presents the results of 25 orthogonal simulation tests designed to support subsequent prediction of surface properties after URCP and optimization of ultrasonic process parameters.

**Table 4:** Orthogonal simulation results of URCP for 42CrMo steel.

Groups	Residual compressive stress (MPa)	Hardness (HRC)	Surface roughness (μm)
1	−1052	59.3	0.248
2	−1098	60	0.217
3	−1132	60.5	0.204
4	−1156	61	0.196
5	−1213	61.2	0.222
6	−1136	60.1	0.197
7	−1172	60.5	0.214
8	−1203	61.1	0.255
9	−1223	60.9	0.268
10	−1217	61	0.242
11	−1232	59.9	0.227
12	−1204	60.4	0.312
13	−1176	61.2	0.306
14	−1195	61.2	0.272
15	−1235	61	0.282
16	−1176	60	0.287
17	−1161	60.9	0.312
18	−1156	60.7	0.285
19	−1173	60.5	0.293
20	−1277	61.2	0.274
21	−1154	59.9	0.282
22	−1166	59.6	0.246
23	−1215	60.7	0.263
24	−1203	61	0.284
25	−1208	61.1	0.284

To further investigate the influence of different URCP parameters on residual compressive stress, surface hardness, and surface roughness of 42CrMo steel—and to better predict and optimize the variation trends and optimal parameter domains of surface properties—additional simulations were conducted with fixed amplitude (7 μm), feed rate (0.05 mm/r), and spindle speed (100 r/min), while varying the static pressure.

#### 2.4.1 Simulated von Mises stress results

**Fig. 6:**
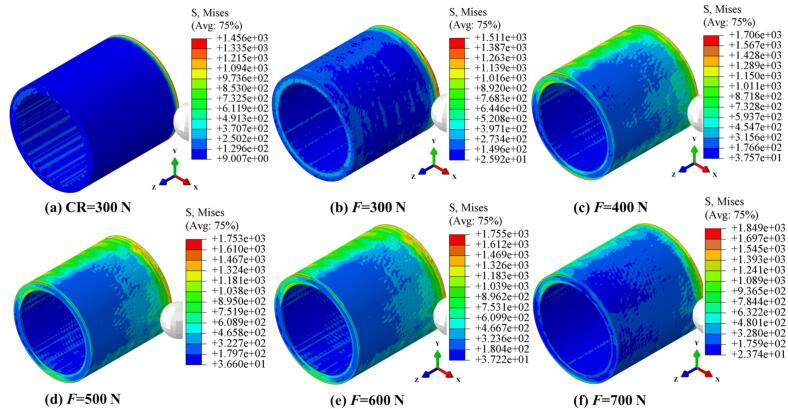
Simulated von Mises stress fields under different static pressures. (a) CR at 300  N; (b)–(f) URCP with static pressures ranging from 300 N to 700 N. (All cases use the same feed rate 0.05 mm/r and spindle speed 100 r/min; amplitude 7 μm is applied only in URCP).

As shown in Fig. 6, the evolution of von Mises stress distribution on the surface of 42CrMo cylindrical specimens under both CR and URCP is compared, with static pressure ranging from 300 N to 700 N. Under CR at 300 N, the stress field induced by the rolling ball is confined to the immediate contact area between the ball and the workpiece, showing a relatively linear and uniform distribution. No significant stress or deformation is observed beyond the contact zone. The maximum von Mises stress under CR is 1456 MPa. In contrast, under URCP, a more pronounced stress gradient field is observed, extending beyond the contact zone due to ultrasonic vibration. Stress waves propagate outward from the contact region between the rolling ball and the workpiece. The minimum von Mises stress increases from 9 MPa under CR to 25.92 MPa under URCP. As the static pressure increases from 300 N to 700 N, the strengthening effect of URCP becomes more prominent, with the maximum von Mises stress rising from 1511 MPa to 1849 MPa, and the minimum stress ranging from 23.74 MPa to 37.72 MPa. Notably, increasing static pressure during URCP not only raises the peak stress values but also expands the affected region, indicating deeper and more extensive plastic deformation compared to CR.

These results confirm that ultrasonic rolling produces a significantly stronger surface strengthening effect than conventional rolling. Moreover, the high-frequency ultrasonic vibration introduces a long-range transmission effect across the entire workpiece. In addition to the static pressure applied by the rolling ball, the high-frequency impact energy radiates from the contact point, inducing stress responses even in non-contact regions. This phenomenon is attributed to the energy transfer and amplification through the ultrasonic generator, transducer, and horn, resulting in high-frequency impact loading. The generated stress waves propagate radially and tangentially through the workpiece, triggering stress responses even in remote, non-contact areas—an effect not observed in CR. This “energy injection” effect of ultrasonic vibration elevates local energy density even in areas untouched by the rolling ball, promoting internal vibration energy in the material. The deeper strengthening layer and broader effective influence zone achieved by URCP not only reflect its enhanced efficiency but also significantly reduce processing energy consumption. As a single pass of URCP can strengthen a wider area, fewer repetitions are required, making URCP a promising technique for green manufacturing by lowering energy use and carbon emissions in actual processing.

**Fig. 7:**
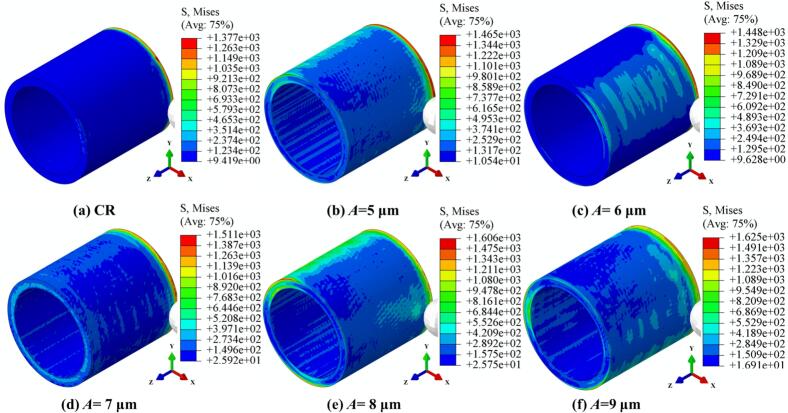
Simulated von Mises stress fields under different amplitudes. (a) CR; (b)–(f) URCP with amplitudes ranging from 5 μm to 9 μm. (All cases use the same feed rate 0.10 mm/r and spindle speed 150 r/min; static pressure is 300 N).

Fig. 7 illustrates the evolution and distribution of von-Mises stress in 42CrMo steel processed by CR and URCP under different amplitudes. In these simulations, the spindle speed was fixed at 150 r/min, the static pressure at 300 N, and the feed rate at 0.10 mm/r. It is observed that CR results in a minimum and maximum von-Mises stress of 9.42 MPa and 1377 MPa, respectively. The stress distribution in CR is highly localized and confined to the contact area between the rolling ball and the workpiece. This is because CR involves only the application of static pressure, without the benefit of ultrasonic energy, and thus cannot induce stress transmission or propagation into the subsurface regions of the 42CrMo steel. As a result, the stress field is restricted to the immediate contact zone. In contrast, Figs. 7b to 7f show the variation of von-Mises stress distribution in URCP with amplitudes ranging from 5 μm to 9 μm. As the amplitude increases, the maximum von-Mises stress rises from 1465 MPa to 1625 MPa, while the minimum stress increases from 10.54 MPa to 16.91 MPa. A higher amplitude expands the stress-affected zone, deepens the residual compressive stress layer, and intensifies plastic deformation. When the material reaches its yield limit, significant plastic deformation occurs, forming a work-hardened layer. This demonstrates that increasing the amplitude enhances the surface strengthening effect of URCP. Notably, Fig. 7c clearly shows that when the amplitude is 6 μm, a rectangular stress zone measuring approximately 30 mm × 15 mm appears behind the rolling ball along the axial direction—even though the ball has not yet physically contacted that region. The boundary of this stress zone exhibits a wave-like contour, a result of the high-frequency ultrasonic vibration transmitted through the material. Unlike CR, which only forms stress at the contact point, URCP generates stress fields not only at the contact area but also ahead and behind the rolling ball, indicating that ultrasonic energy and stress waves are transmitted and radiated within the 42CrMo steel. This significantly broadens the effective strengthening zone, enhances surface performance, and increases processing efficiency while reducing the number of required passes, aligning with the goals of efficient and green manufacturing.

**Fig. 8:**
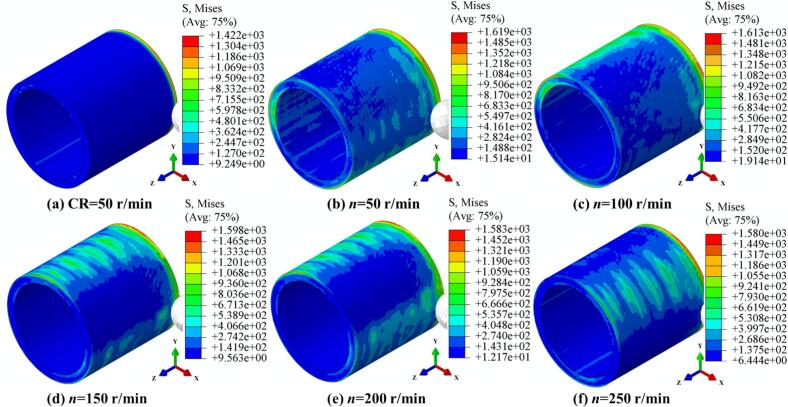
Simulated von Mises stress fields under different spindle speeds. (a) CR at 50 r/min; (b)–(f) URCP with spindle speeds ranging from 50 r/min to 250 r/min (All cases use the same feed rate 0.15 mm/r and static pressure 700 N; amplitude 5 μm is applied only in URCP).

Fig. 8 presents the von-Mises stress evolution and distribution under a constant static pressure of 700 N, amplitude of 5 μm, and feed rate of 0.15 mm/r, with varying spindle speeds during both CR and URCP. The spindle speed was varied from 50 r/min to 250 r/min in increments of 50 r/min. As the spindle speed increased, the maximum von-Mises stress gradually decreased from 1619 MPa to 1580 MPa, and the minimum stress decreased from 15.14 MPa to 6.44 MPa. Nonetheless, both the maximum and minimum stress values in URCP remained higher than those in CR, reaffirming the effectiveness of ultrasonic energy in transmitting and dispersing stress within the material. From the stress distributions at 100 r/min, 150 r/min, 200 r/min, and 250 r/min, it is evident that even in regions not yet reached by the rolling ball, stress waves induced by ultrasonic vibration are present—typically forming a rectangular zone of about 30 mm × 18 mm. These stress waves propagate radially and axially, enhancing the strengthening effect. However, it should be noted that as spindle speed increases, the maximum von-Mises stress decreases, suggesting a reduction in hardening intensity. This implies that lower spindle speeds can produce more pronounced stress fields and better strengthening effects. Moreover, since the URCP model incorporates thermo-mechanical coupling, increased spindle speeds lead to higher temperatures in the contact region, which reduces the yield strength of 42CrMo steel. This makes the material more prone to plastic deformation, resulting in surface flow rather than accumulating higher stresses. Excessively high spindle speeds also reduce the energy coupling efficiency between the ultrasonic transducer and the workpiece, making it difficult to achieve optimal resonance. As a result, ultrasonic energy transmission and penetration are weakened, the superposition of frequency and amplitude becomes unstable, and the dynamic impact effect of ultrasonic vibration is diminished, leading to a reduction in plastic deformation and overall strengthening performance.

**Fig. 9:**
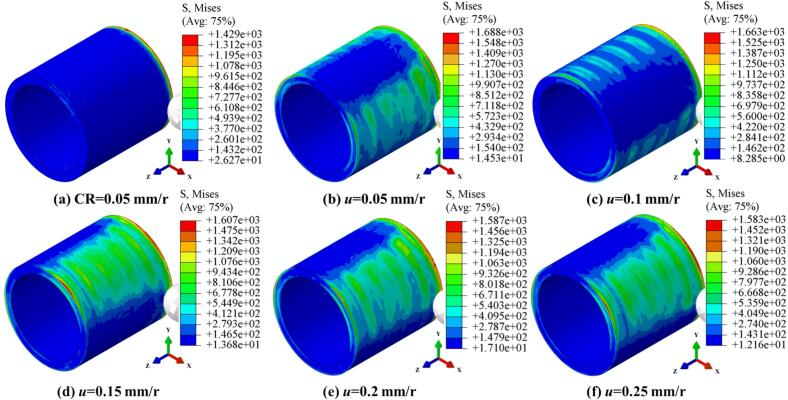
Simulated von Mises stress fields under different feed rates. (a) CR at 0.05 mm/r; (b)–(f) URCP with feed rates ranging from 0.05 mm/r to 0.25 mm/r (All cases use the same spindle speed 50 r/min and static pressure 400 N; amplitude 7 μm is applied only in URCP).

Fig. 9 illustrates the evolution and distribution of von-Mises stress in CR and URCP processes under varying feed rates, while maintaining a constant spindle speed of 50 r/min, static pressure of 400 N, and amplitude of 7 μm. In the CR process, stress is limited strictly to the contact area between the rolling ball and the workpiece, with no stress propagation or ultrasonic energy transmission outside the contact zone. In contrast, Figs. 9b to 9f demonstrate that with ultrasonic excitation, stress fields are generated not only in the contact area but also in the regions ahead of the rolling ball. As the feed rate increases from 0.05 mm/r to 0.25 mm/r, the maximum von-Mises stress decreases from 1688 MPa to 1583 MPa, and the minimum stress fluctuates between 14.53 MPa and 8.25 MPa. Although the minimum stress values in URCP are sometimes lower than those in CR, the stress propagation range is significantly wider in URCP. Unlike CR, where stress is concentrated in a narrow region, URCP introduces additional dynamic loading through high-frequency vibrations, inducing micro-scale impacts over a broader area and promoting microplastic deformation. Ultrasonic waves are continuously generated and transmitted from the contact point, expanding the influence zone of the equivalent stress. Despite localized decreases in minimum stress, URCP results in a more uniform and deeper distribution of plastic strain energy across the workpiece, thereby increasing the depth and effectiveness of the surface strengthening process.

#### 2.4.2 Temperature rise results

**Fig. 10:**
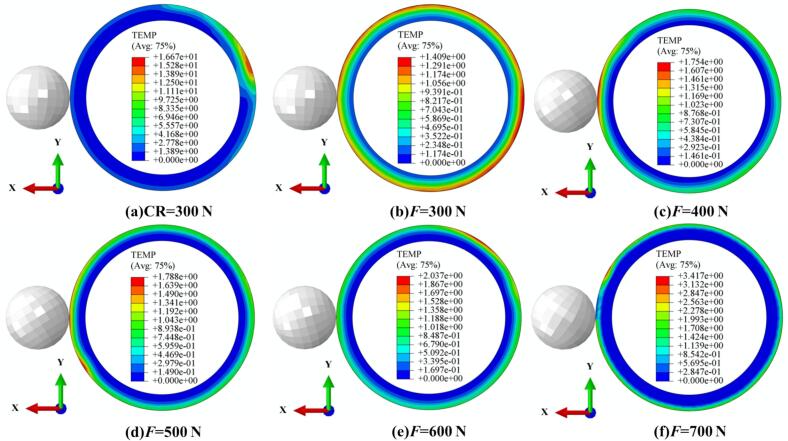
Simulated thermal response of 42CrMo steel under different static pressures. (a) CR at 300 N; (b)–(f) URCP with static pressures ranging from 300 N to 700 N. (All cases use the same feed rate 0.05 mm/r and spindle speed 100 r/min; amplitude 7 μm is applied only in URCP).

To simultaneously illustrate the temperature rise in the thermomechanically coupled URCP simulation model, the temperature variations under different static pressures, amplitudes, spindle speeds, and feed rates were analyzed. As shown in Fig. 10, the temperature increases during CR and URCP at various static pressures are presented. In the URCP simulations, static pressure varied from 300 N to 700 N. At an ambient temperature of 20 °C, the maximum absolute temperature rise in CR reached 16.7 °C. In contrast, the temperature rise in URCP was limited to within 5 °C. Specifically, Fig. 10b shows that under a static pressure of 300  N, the temperature increased by only 1.4 °C. As the static pressure increased, the maximum temperature rise also gradually increased—from 1.7 °C at 400  N to 3.4 °C at 700  N. This confirms that the temperature rise during URCP is significantly lower than that of CR. The lower thermal load in URCP is attributed to its working mechanism: traditional rolling relies solely on mechanical extrusion and frictional heat, with prolonged contact time and high contact pressure, leading to intense plastic deformation and prominent heat accumulation in the contact zone. In contrast, URCP introduces ultrasonic energy into the traditional process via high-frequency vibrations. These vibrations impose intermittent loading, leading to more uniform contact stress distribution within a unit of time. This helps reduce both frictional heating and heat accumulation from plastic deformation. Moreover, the energy generated by the ultrasonic transducer primarily serves to activate the dislocation energy within 42CrMo steel, promoting lattice slip rather than being converted into excessive heat. The high-frequency ultrasonic vibrations reduce flow stress, enabling easier plastic deformation and lowering frictional forces during processing. As a result, heat generated from friction under high resistance is effectively minimized. Compared with CR, the contact duration per unit area between the rolling ball and the workpiece is longer, contributing to more frictional heat. The high-frequency vibration in URCP creates intermittent gaps between the rolling ball and workpiece, decreasing both heat transfer and accumulation. Additionally, this vibration improves surface convective heat transfer, promoting the dissipation of heat. Micro-gaps and a disturbed vibration layer form on the surface, allowing lubricant and air to continuously enter the contact zone, effectively achieving cooling and preventing the formation of localized hot spots.

**Fig. 11:**
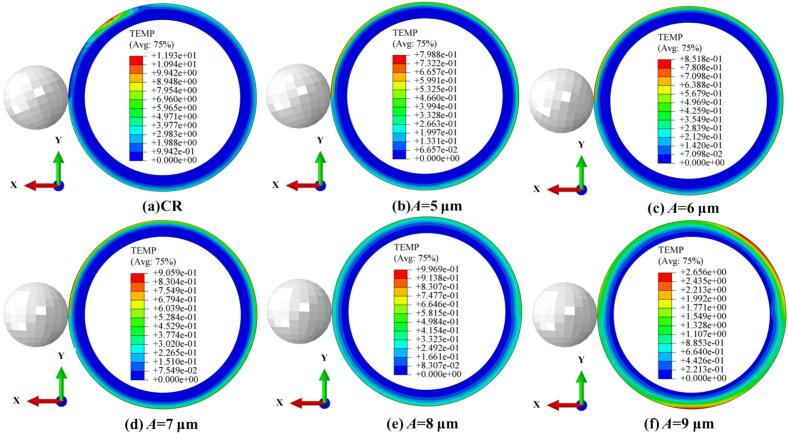
Simulated thermal response of 42CrMo steel under different amplitudes. (a) CR; (b)–(f) URCP with amplitudes ranging from 5 μm to 9 μm. (All cases use same feed rate 0.10 mm/r and spindle speed 150 r/min; static pressure is 300 N).

As shown in Fig. 11, temperature changes under different amplitudes during CR and URCP are illustrated. Fig. 11a clearly shows that during CR, the surface temperature increased by approximately 12 °C from room temperature. Figs. 11b to 11f show the temperature variation as amplitude increases from 5  μm to 9  μm. It can be seen that the absolute temperature rise increases with amplitude—from 0.8 °C to 2.6 °C. Although higher amplitudes lead to slightly higher processing temperatures, Fig. 11f demonstrates that the temperature distribution becomes more uniform at 9  μm amplitude. A structured thermal gradient layer forms, enabling effective distribution and diffusion of thermal stress. Still, compared with CR, the overall temperature rise remains much lower. This reduction is due to the unique high-frequency vibration mechanism in URCP, which disrupts the continuous contact model seen in CR and forms intermittent thermal contact. Consequently, the accumulation of frictional and plastic deformation heat per unit time is substantially reduced. Additionally, ultrasonic vibrations activate dislocation energy and lattice slip within 42CrMo steel, reducing flow stress and achieving more uniform plastic deformation, while improving heat dissipation at the material surface. Thus, URCP enables not only surface property enhancement but also lower energy consumption and thermal load, highlighting its green and efficient processing advantages.

**Fig. 12:**
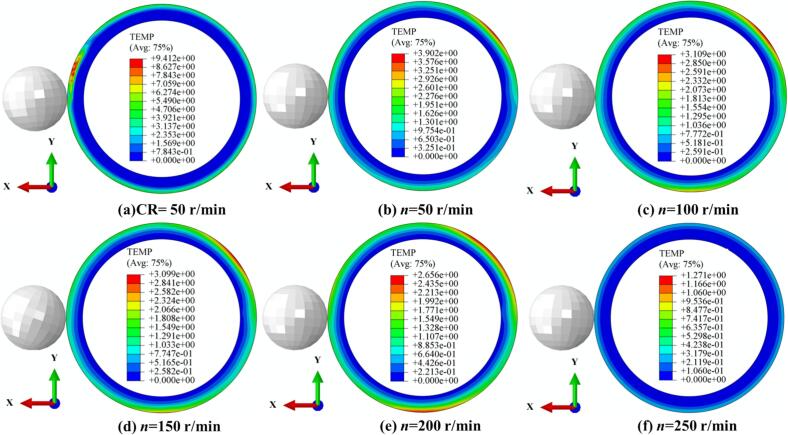
Simulated thermal response of 42CrMo steel under different spindle speeds. (a) CR at 50 r/min; (b)–(f) URCP with spindle speeds ranging from 50 r/min to 250 r/min. (All cases use the same feed rate 0.15 mm/r and static pressure 700 N; amplitude 5 μm is applied only in URCP).

Fig. 12 shows the temperature changes during CR and URCP at different spindle speeds. As seen in Fig. 12a, CR results in a maximum temperature rise of 9.4 °C, with localized heat concentration in the processing zone. Figs. 12b to 12f depict the temperature variations in URCP as spindle speed increases from 50 r/min to 250 r/min. At 50 r/min, the maximum temperature rise reaches 3.9 °C, whereas at 250 r/min, it drops to just 1.3 °C. Notably, from 50 r/min to 150 r/min, the reduction in surface temperature is relatively small (3.9 °C to 3.1 °C). However, when the speed reaches 200 r/min and 250 r/min, the temperature decreases more significantly (down to 2.6 °C and 1.3 °C, respectively). This suggests that at higher spindle speeds, the contact time between the ball and the workpiece shortens, limiting heat accumulation and enabling faster heat dissipation. At low speeds, although ultrasonic vibration reduces friction and promotes separation, the prolonged contact still causes greater plastic deformation and heat generation. Nonetheless, the total heat generated during URCP is much lower than that of CR. From an energy perspective, all processing follows the principle of energy conservation. The significantly lower temperature rise in URCP indicates that the ultrasonic energy is not primarily converted into heat, but more efficiently transformed into mechanical energy driving plastic deformation. This energy utilization pattern minimizes thermal waste and enhances green manufacturing efficiency. More importantly, the energy is concentrated on modifying the material’s microstructure, improving surface residual stress, hardness, and surface roughness—showcasing URCP’s advantages in strengthening efficiency and energy utilization.

**Fig. 13:**
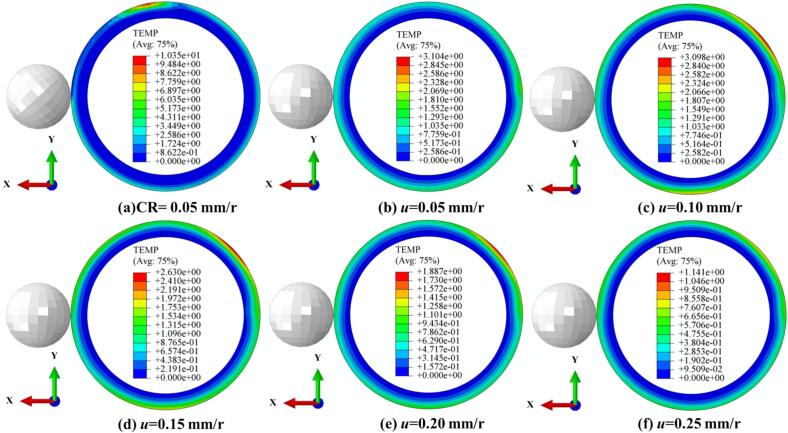
Simulated thermal response of 42CrMo steel under different feed rates. (a) CR at 0.05 mm/r; (b)–(f) URCP with feed rates ranging from 0.05 mm/r to 0.25 mm/r. (All cases use the same spindle speed 50 r/min and static pressure 400 N; amplitude 7 μm is applied only in URCP).

As shown in Fig. 13, the temperature increase trends under various processing parameters during CR and URCP are compared. In Fig. 13a, the surface temperature in CR rises by 10.2 °C. Figs. 13b to 13f show the temperature variations at different feed rates during URCP. At a feed rate of 0.05  mm/r, the absolute temperature rise is highest at 3.1 °C. As the feed rate increases, the temperature gradually decreases. When the feed rate reaches 0.10  mm/r, the temperature stabilizes around 3.0 °C. At 0.15  mm/r and 0.20  mm/r, the temperature drops further to 2.6 °C and 1.8 °C, respectively, reaching its lowest at 0.25  mm/r. This confirms the critical role of feed rate in URCP. On one hand, higher feed rates reduce the contact time between the rolling ball and the workpiece per unit time, leading to less heat conduction and lower temperature rise. On the other hand, the combination of high-frequency ultrasonic impact and high feed rate facilitates rapid heat dissipation, suppressing local heat accumulation. Microscopically, ultrasonic energy is primarily transformed into plastic deformation energy of the surface grains, manifested as dislocation movement and lattice distortion, with only a small portion converted into heat. Although lower feed rates slightly increase the processing temperature, the extent of this rise is still much smaller than that in CR. At higher feed rates, ultrasonic excitation enhances heat dispersion, reducing heat accumulation and resulting in lower temperature rise. Overall, the temperature variation trends during CR and URCP confirm that CR—based on rigid mechanical contact and extrusion—has low energy transfer efficiency, with most mechanical energy lost as heat. In contrast, URCP, as a green, efficient, and low-carbon process, minimizes heat accumulation regardless of feed rate. Ultrasonic vibrations promote heat dissipation and prevent excessive frictional heating. Consequently, more energy is directed toward plastic deformation of the material, effectively enhancing surface properties of 42CrMo steel while achieving green and efficient processing.

#### 2.4.3 Deep residual stress results

**Fig. 14:**
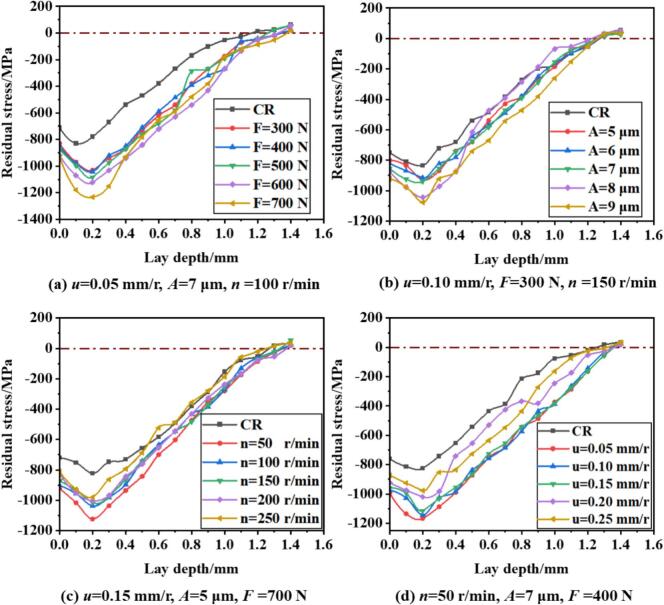
Variation of residual stress with depth under different parameters in URCP and CR.

To further investigate the residual stress distribution along the depth of the material under URCP and CR strengthening, the residual stress profiles are plotted in Figs. 14a to 14d. Fig. 14a clearly illustrates the distribution of residual stress at various static pressures. It can be observed that the maximum compressive residual stress after URCP occurs at a depth of approximately 0.2 mm from the surface. As the depth increases, the compressive residual stress decreases gradually after reaching its peak. At a depth of 1.4 mm, the compressive residual stress transitions into tensile residual stress. With increasing static pressure, the maximum compressive residual stress also increases. When the static pressure reaches 700 N, the maximum compressive residual stress peaks at –1233 MPa. In contrast, at a static pressure of 300 N, the peak stress is only –1030 MPa. Notably, increasing the static pressure from 300 N to 600 N results in a relatively modest improvement in compressive residual stress (from –1030 MPa to –1123 MPa), while the increase from 600 N to 700 N leads to a more significant enhancement. During conventional rolling, the peak compressive residual stress is located closer to the surface, and the entire residual stress profile is shifted upward. At a depth of around 0.1 mm, the peak compressive residual stress is only –830 MPa. This comparison demonstrates that URCP produces a significantly stronger compressive residual stress field than CR. This is because CR involves static loading, which leads to a linearly stable stress state that cannot effectively propagate deep into the material. Consequently, the residual stress is mainly confined to the indentation and contact region. In contrast, the dynamic impact loading induced by ultrasonic vibration in URCP allows the ultrasonic energy to penetrate not only beneath the rolling ball but also to surrounding areas. The energy disperses through the material due to the excitation of the system's natural frequency, resulting in a deeper and broader plastically deformed layer. Therefore, even regions not in direct contact with the rolling ball can develop compressive residual stress. The dynamic loading effect of URCP can be viewed as a periodic loading–unloading process. This unique feature of ultrasonic excitation helps overcome the inherent yield resistance of high-strength steels, facilitating the introduction of residual compressive stress into the material surface and subsurface. From a microstructural perspective, URCP promotes grain refinement and dislocation strengthening in the surface layer of 42CrMo steel, enhancing the strengthening effect on the microstructural level. On the other hand, CR relies solely on pure mechanical pressing. As a result, not only is the residual stress field produced by CR shallower and weaker, but URCP also yields a stronger, more stable, and longer-lasting residual stress field with a broader influence range. This highlights the high efficiency and eco-friendliness of URCP, which significantly improves the surface properties of metallic materials. With lower energy input, efficient and sustainable surface strengthening can be achieved.

Fig. 14b further shows the residual stress distribution profiles under CR and URCP at different ultrasonic amplitudes. As the amplitude increases, the peak residual compressive stress increases noticeably. It is observed that at amplitudes of 5 μm, 6 μm, and 7 μm, the maximum residual compressive stress fluctuates between –930 MPa and –940 MPa. When the amplitude increases to 8 μm and 9 μm, the stress jumps significantly, reaching values between –1040 MPa and –1077 MPa, clearly higher than those at lower amplitudes. This is because higher amplitudes deliver more kinetic energy to the surface per unit time. In fact, energy input in URCP is directly proportional to the ultrasonic amplitude [39]; larger amplitudes generate stronger excitation energy, which promotes more intense plastic deformation. Simultaneously, the increased dislocation density and work hardening under URCP amplify the effect on residual stress. Therefore, as amplitude increases, the maximum residual compressive stress also rises. In contrast, CR, which lacks ultrasonic excitation, produces a peak residual compressive stress of only –835 MPa. Residual stress originates from the imbalance of internal microstructures caused by plastic deformation—while the inner elastic zone attempts to recover, the outer hardened layer resists, generating a residual compressive stress field and hardened layer. This means that greater plastic deformation and higher hardening lead to stronger elastic recovery forces, which in turn generate larger compressive residual stresses to maintain internal stress equilibrium and structural stability. Although each 1 μm increase in amplitude appears small, when the amplitude exceeds a certain threshold (around 8 μm), the excitation frequency of the ultrasonic system achieves a more ideal resonance coupling condition. At amplitudes of 8 μm and 9 μm, the material enters a “critical activation zone” in terms of energy response, leading to a dramatic enhancement in its responsiveness to ultrasonic energy and a sharp rise in residual compressive stress. For 42CrMo steel, this indicates that 8 μm represents a threshold amplitude for an effective ultrasonic response. Below this threshold, the energy induced by URCP is insufficient to generate a deeper residual stress layer. Once this threshold is exceeded, a significant stress accumulation effect occurs. Although amplitudes in the range of 5–7 μm do induce some strengthening, they mainly serve as auxiliary reinforcement. The excitation at these amplitudes is not strong enough to reach the maximum plastic deformation zone, resulting in no evident nonlinear surge in residual stress.

Regarding the variations in residual stress fields under CR and URCP at different workpiece rotation speeds, as shown in Fig. 14c, the results clearly illustrate how residual stresses change as the rotation speed increases from 50 r/min to 250 r/min. The maximum residual compressive stress at a depth of 0.2 mm reaches only –823 MPa under CR, which is significantly lower than that achieved under URCP. At a rotation speed of 250 r/min, the maximum residual compressive stress produced by URCP is –980 MPa. As the rotation speed decreases, the maximum residual compressive stress under URCP increases. Specifically, when the rotation speed is gradually reduced from 250 r/min to 200, 150, and 100 r/min, the maximum residual compressive stress increases from –980 MPa to –1003 MPa, –1015 MPa, and –1037 MPa, respectively. When the rotation speed further decreases to 50 r/min, the peak residual compressive stress reaches a maximum of –1124 MPa. This trend can be attributed to the shortened contact time between the rolling ball and the workpiece surface at higher rotation speeds. Consequently, the ultrasonic energy cannot be sufficiently transmitted and accumulated on the material surface, leading to a reduced degree of plastic deformation. As a result, the residual compressive stress induced by URCP is weakened, and the maximum stress value decreases. Moreover, the strengthening mechanism of URCP heavily depends on the resonant coupling state between the high-frequency excitation and the workpiece surface. At excessively high rotation speeds, the contact point between the ultrasonic tool and the material changes rapidly, making it difficult to maintain stable coupling and generate reinforcing ultrasonic waves. This reduces the efficiency of ultrasonic energy utilization, thereby hindering the effective deposition of residual compressive stress in the surface layer. From the perspective of friction and heat generation during URCP, high rotation speeds may lead to sliding friction rather than rolling friction between the rolling ball and the workpiece, thereby causing localized heat generation [40]. This thermal softening effect partially offsets the work hardening effect, indirectly diminishing the retention of residual compressive stress. In contrast, when the rotation speed is set to a low value such as 50 r/min, the contact time between the rolling ball and the workpiece surface is significantly extended. With sufficient strengthening time, plastic deformation in the material is more effectively accumulated. The ultrasonic vibrations can continuously impact the contact area at high frequency and for longer durations, promoting deeper plastic deformation. The 42CrMo steel effectively responds to this ultrasonic impact within such an “optimal strengthening window,” resulting in a greater accumulation of residual compressive stress. Additionally, lower rotation speeds enhance the dynamic stability of the ultrasonic strengthening system, reducing eccentricity and vibration. This facilitates the formation of ideal ultrasonic resonance coupling and improves the efficiency of ultrasonic energy conversion, ultimately yielding significantly higher residual compressive stress values.

Fig. 14d illustrates the depth-wise distribution of residual compressive stress under different feed rates for both CR and URCP. Under CR, the maximum residual compressive stress at a depth of 0.2 mm is only –824 MPa. With ultrasonic energy applied and the feed rate set to 0.25 mm/r, the peak stress increases to –977 MPa. As the feed rate decreases further to 0.20 mm/r, the maximum residual stress rises to –1020 MPa. At a feed rate of 0.15 mm/r, the stress increases more significantly, reaching –1115 MPa. In other words, as the feed rate decreases, the maximum residual compressive stress increases. However, the rate of increase in stress is not uniform. Between 0.25 mm/r and 0.20 mm/r, the increase is relatively modest. In contrast, between 0.20 mm/r and 0.15 mm/r, the increase is more pronounced. When the feed rate decreases from 0.15 mm/r to 0.10 mm/r, the change in residual stress is minor, rising only from –1115 MPa to –1143 MPa. At the lowest feed rate of 0.05 mm/r, the maximum residual compressive stress reaches the highest value observed across all experimental conditions, namely –1167 MPa. This is because lower feed rates extend the interaction time between the rolling ball and each unit length of the workpiece. As a result, the surface experiences a longer duration of ultrasonic energy impact and plastic deformation, increasing the residual compressive stress. When the feed rate drops from 0.20 mm/r to 0.15 mm/r, the surface of the workpiece appears to surpass a threshold of accumulated ultrasonic and mechanical action. In this range, the material surface exhibits a highly responsive zone, or “sensitive zone,” for plastic deformation, resulting in a steeper increase in residual stress. During this stage, the coupling between ultrasonic excitation and the plastic hardening mechanism of 42CrMo steel is most effective. However, as the feed rate continues to decrease, although the contact duration per unit length further increases, the material's strengthening effect approaches saturation. From a microstructural perspective, the grain refinement, dislocation density, and surface compaction may reach their limits, making further significant increases in residual compressive stress difficult. More importantly, excessively long processing times at very low feed rates may trigger thermal effects or recovery processes at the microscale, which can cause partial stress release or rearrangement. This ultimately limits the effectiveness of further ultrasonic rolling strengthening. Therefore, a reasonable feed rate must be selected to optimize the surface performance enhancement in URCP.

#### 2.4.4 Hardness results

**Fig. 15:**
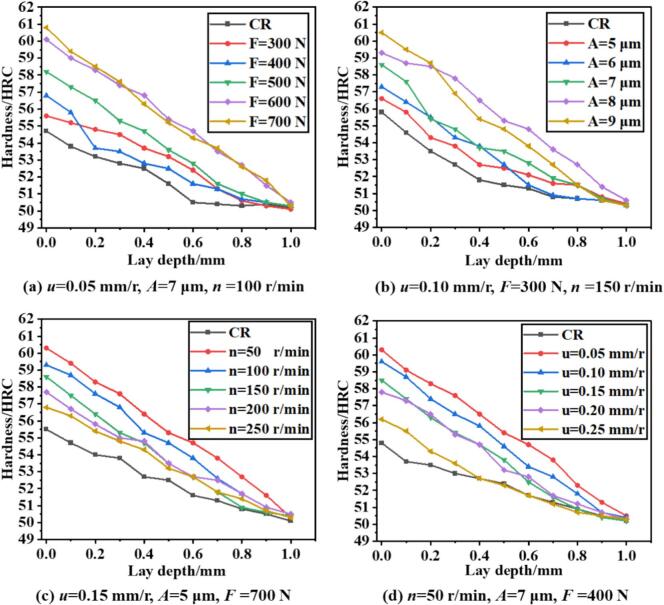
Variation of surface hardness with depth under different parameters in URCP and CR.

Fig. 15 presents the distribution and evolution of surface hardness with depth under different processing parameters, comparing CR and URCP. Fig. 15a illustrates the hardness distribution under varying static pressures for both CR and URCP. Without ultrasonic vibration, the surface Rockwell hardness of the material is only 54.7 HRC—approximately 4.7 HRC higher than the base material's hardness of 50 HRC. When ultrasonic rolling is applied at a static pressure of 300 N, the surface hardness increases to 55.6 HRC, significantly higher than in the absence of ultrasonic vibration. In both CR and URCP, surface hardness gradually decreases with increasing depth until it levels off at the base hardness around a depth of 1 mm. Furthermore, with increasing static pressure, surface hardness also increases. At 700 N, the surface hardness reaches the maximum value observed in all test conditions—60.8 HRC. For static pressures of 400 N, 500 N, and 600 N, the hardness increases approximately linearly to 56.8 HRC, 58.2 HRC, and 60.1 HRC, respectively. However, when static pressure increases from 600 N to 700 N, the surface hardness increases by only 0.7 HRC, indicating a diminishing return. These results suggest that higher static pressure leads to more intense surface plastic deformation, resulting in higher hardness. As static pressure increases, the stress exerted by the rolling ball on the surface intensifies, thereby increasing the plastic strain within a unit volume. On the microscopic scale, this causes severe grain compression, increased dislocation density, and the formation of new textures. These mechanisms enhance the strain hardening effect of URCP, leading to a higher surface hardness. In the pressure range from 300 N to 600 N, surface hardening exhibits a linear growth trend, representing a high-sensitivity phase. During this stage, the rolling energy is primarily concentrated on the surface layer, and work hardening progressively accumulates, causing a rapid increase in surface hardness. The material's plastic potential has not yet been fully exhausted, hence the pronounced change in hardness. However, between 600 N and 700 N, grain refinement reaches a high degree and the stress state in the surface region stabilizes, meaning work hardening approaches saturation. Further increases in static pressure no longer significantly enhance hardness. Additionally, excessive static pressure may lead to localized elastic recovery or stress redistribution, preventing the additional load from being fully converted into effective plastic deformation. Overly high pressure can also cause localized temperature rise and dislocation rearrangement, which further suppress the accumulation of strain hardening and slow down the hardness increase.

Fig. 15b clearly shows the surface hardness distribution with depth under various ultrasonic amplitudes in URCP, alongside results for CR. Without ultrasonic vibration, the surface hardness reaches the lowest value among all tested conditions—only 55.8 HRC. With a 5 μm amplitude, surface hardness increases to 56.6 HRC. As the amplitude increases from 5 μm to 6 μm, hardness rises to 57.3 HRC, and further increases to 58.6 HRC at 7 μm. At 8 μm, the surface hardness peaks at 60.5 HRC under a static pressure of 700 N. This trend suggests that higher amplitudes lead to stronger ultrasonic energy input, thus enhancing the work hardening effect. As amplitude increases, the vibrational displacement during the rolling-ball–surface interaction also grows, increasing ultrasonic energy input per unit time. Combined with the static pressure from traditional rolling, the ultrasonic vibration contributes to stronger dynamic impacts, intensifying micro-plastic deformation and ultrasonic shockwave excitation. This promotes the proliferation and entanglement of dislocations, thus improving the surface hardening. Moreover, high amplitude results in more complex and severe stress–strain states at the surface. A high-density dislocation network and substructure formation significantly enhance strain hardening. Dynamic recrystallization and grain fragmentation during this process further contribute to increased surface hardness. Compared with conventional rolling, ultrasonic rolling integrates static pressure with ultrasonic vibration, enhancing the synergistic effect between the two. Under high amplitude and static pressure, a composite deformation zone is formed, where greater plastic deformation can occur over larger depths and areas, thereby markedly increasing surface hardness.

Fig. 15c depicts the surface hardness distribution under different workpiece rotational speeds for CR and URCP. Under CR conditions (static pressure only), the surface hardness reaches 55.5 HRC. With URCP applied, hardness is significantly improved. At 250 r/min, surface hardness reaches 56.8 HRC. As rotational speed decreases, the hardness of 42CrMo steel increases. At 50 r/min, hardness peaks at 60.3 HRC. Specifically, as speed decreases from 250 r/min to 200, 150, and 100 r/min, surface hardness increases by 0.9 HRC, 1.8 HRC, and 2.5 HRC, respectively. This is because lower rotational speeds result in longer contact time between the rolling ball and the surface per unit length, increasing the number of ultrasonic impacts each surface point receives. Consequently, the material undergoes more extensive plastic deformation, leading to enhanced strain hardening and elevated hardness. In contrast, at higher speeds, the material quickly moves away from the vibration zone, lowering ultrasonic energy coupling efficiency. At lower speeds, the interaction between surface movement and ultrasonic frequency becomes more synchronized, improving energy transmission efficiency and the conversion of ultrasonic energy into the mechanical work required for plastic deformation. This further enhances surface hardness. In CR or high-speed URCP conditions, more heat is generated, possibly causing thermal softening and stress recovery. Lower rotational speeds reduce heat generation, preserving the hardened state after plastic deformation and resulting in a more stable and harder surface.

Fig. 15d shows the influence of feed rate on the surface hardness of 42CrMo steel under CR and URCP. With only static pressure applied, CR increases surface hardness to 54.8 HRC. Under URCP with a feed rate of 0.25 mm/r, hardness improves to 56.2 HRC. As the feed rate decreases, surface hardness continues to increase, reaching a maximum of 60.3 HRC at 0.05 mm/r—a 10.3 HRC improvement over the base material. Lower feed rates allow the rolling ball to remain in contact with each surface point for a longer time, increasing the frequency of contact and ultrasonic impacts. This leads to more pronounced plastic deformation, higher dislocation density, and enhanced work hardening, thereby raising surface hardness. Additionally, ultrasonic energy needs time to transfer into the material. At higher feed rates, the rolling ball moves too quickly for effective energy transfer. Reducing the feed rate concentrates ultrasonic energy more efficiently, increasing plastic deformation and strengthening. However, the hardness increase becomes less pronounced when the feed rate decreases from 0.10 mm/r to 0.05 mm/r, indicating a saturation effect in strain hardening. At low feed rates, each surface unit has already received sufficient ultrasonic energy, and further reduction does not significantly improve hardness. Excessively low feed rates also reduce processing efficiency, which is counterproductive to high-efficiency manufacturing. Moreover, as a high-strength steel, 42CrMo has a limit to its surface strengthening. Continued deformation leads to dislocation saturation, making further plastic deformation increasingly difficult, thereby limiting further hardness improvement. In such cases, grain refinement and reorientation show diminishing returns on hardness enhancement.

#### 2.4.5 Average surface roughness results

**Fig. 16:**
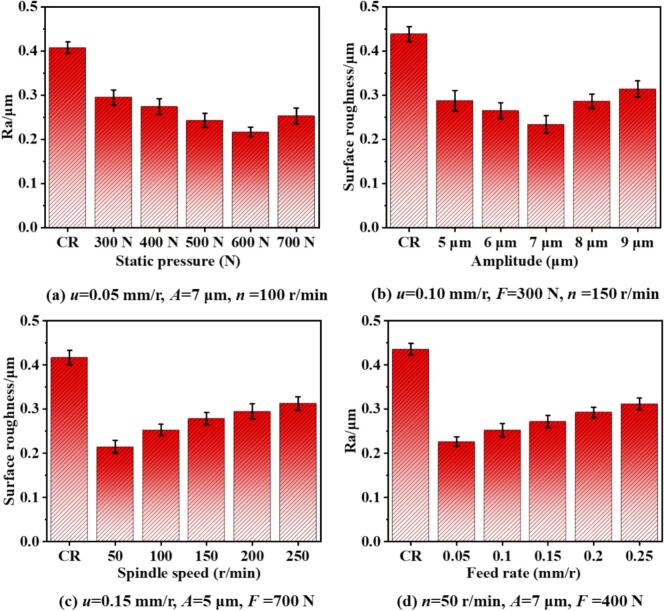
Variation of surface roughness under different parameters in URCP and CR.

Fig. 16 illustrates the variation in average surface roughness (Ra) of 42CrMo steel under CR and URCP with different processing parameters. As shown in Fig. 16a, the surface roughness under different static pressures was analyzed. Under conventional rolling, the surface roughness reached 0.407 μm. When ultrasonic vibration was superimposed and a static pressure of 300 N was applied, the surface roughness decreased to 0.295 μm. This indicates that URCP can significantly reduce surface roughness compared to CR. Moreover, in URCP, the surface roughness first decreases and then increases with increasing static pressure. The lowest roughness of 0.217 μm was achieved at 600 N, whereas further increasing the pressure to 700 N caused roughness to rise to 0.253 μm. Between 300 N and 600 N, surface roughness gradually decreased: 0.295 μm at 400 N and 0.243 μm at 500 N. This non-monotonic trend suggests a nonlinear relationship between static pressure and surface roughness. As the static pressure increases, the contact force between the rolling ball and the material intensifies, flattening surface asperities and densifying the surface. Meanwhile, greater pressure enhances ultrasonic impact energy, promoting deeper plastic deformation and more uniform surface morphology. However, excessive static pressure beyond 600 N may lead to surface tearing, material accumulation, or even reverse flow. Under such conditions, excess material may overflow from the contact region, forming microcracks or pile-ups elsewhere. Additionally, high pressure may hinder uniform ultrasonic energy transmission, introducing micro-disturbances that counteract surface smoothing and cause roughness to rebound.

Fig. 16b presents average surface roughness variations under different ultrasonic amplitudes. The highest roughness, 0.438 μm, was again observed under conventional rolling. When the amplitude was set to 5 μm, roughness dropped to 0.287 μm. As the amplitude increased further, surface roughness initially decreased and then increased. The minimum value, 0.234 μm, was observed at 7 μm. Within the 5–7 μm range, roughness showed a downward trend, reaching 0.265 μm at 6 μm. However, increasing amplitude from 7 μm to 9 μm caused roughness to rise again: 0.286 μm at 8 μm and 0.314 μm at 9 μm. Larger amplitudes intensify energy input at the contact interface, enhancing the ability of ultrasonic impacts to break down and flatten surface protrusions. This results in a denser and smoother surface. However, excessive amplitude can lead to instability, causing local surface waviness or micro-delamination. Uneven plastic flow and phenomena such as “rebound” or “material piling” may occur. Furthermore, the effective contact time between the rolling ball and workpiece decreases under large amplitudes, disrupting energy transfer and diminishing the smoothing effect.

Fig. 16c depicts the effects of workpiece rotational speed on surface roughness. Under CR, roughness peaked at 0.417 μm. In contrast, the lowest roughness of 0.215 μm was achieved under URCP. However, increasing the rotational speed in URCP led to increased roughness. At 250 r/min, roughness reached a maximum of 0.313 μm, significantly higher than that at lower speeds and even higher than CR. As the speed increased from 50 r/min to 250 r/min (passing through 100, 150, and 200 r/min), surface roughness rose from 0.253 μm to 0.279 μm and 0.313 μm, respectively. Higher speeds reduced the contact time per unit area, limiting plastic deformation and preventing full flattening of micro-asperities. Moreover, faster relative motion decreases ultrasonic energy coupling efficiency, leading to coordination mismatch between vibration cycles, rotation, and feed motion. This can produce skipping effects, non-uniform indentations, vibration marks, or drag marks. Additionally, higher speeds increase friction-induced local temperature rises, potentially softening the surface and causing rebound, thus degrading the flattening effect and increasing roughness.

Fig. 16d shows the effect of feed rate on surface roughness. In URCP, roughness gradually increased with feed rate. The lowest value of 0.227 μm was observed at 0.05 mm/r, and the highest (0.312 μm) at 0.25 mm/r—still lower than the 0.436 μm of CR. At intermediate feed rates of 0.10, 0.15, and 0.20 mm/r, the surface roughness values were 0.227 μm, 0.253 μm, and 0.272 μm, respectively. With increasing feed rate, the rolling ball spends less time per unit length, reducing the number of compaction cycles and weakening plastic deformation. Since URCP relies on combined rolling pressure and ultrasonic excitation to achieve plastic flow and micro-smoothing, insufficient interaction time under high feed rates hampers this effect, leading to micro-pits or residual grooves. Moreover, high feed rates reduce the effectiveness of ultrasonic energy application, impairing the polishing and flattening effect. Nevertheless, even at higher feed rates, URCP consistently outperformed CR in reducing surface roughness. This is due to the high-frequency (28 kHz) ultrasonic vibration promoting grain slip, diffusion, and rearrangement, enhancing surface flattening. Ultrasonic vibration also disrupts stress concentrations at the tool–workpiece interface, preventing surface defects such as tearing and scoring. It introduces cavitation-like or micro-lifting effects that reduce frictional resistance, minimizing scratch-induced roughness.

In summary, ultrasonic rolling of 42CrMo steel significantly enhances residual compressive stress distribution in both radial and axial directions compared to conventional rolling, which affects only the subsurface region directly beneath the ball. URCP not only improves residual stress and hardness but also yields superior surface roughness. However, increased residual stress and hardness often correlate with increased roughness. To achieve optimal synergy between surface quality and performance, multi-objective optimization algorithms should be adopted to determine the most effective processing parameter window, aligning with high-efficiency, green, and low-emission manufacturing goals.

## 3 URCP experiments

### 3.1 Materials

URCP was carried out on 42CrMo steel rods. The material was heat treated by high-temperature quenching at 850 °C to obtain a hard martensitic microstructure for 1 h. The quenching medium used was a factory-specified oil-based polymer quenchant. Following quenching, low-temperature tempering at 180 °C was conducted to relieve thermal stresses, reduce the likelihood of surface cracks caused by thermal treatment, lower brittleness, and improve both hardness and toughness of the material. The 42CrMo steel rods used in the experiments had a diameter of 30 mm and a length of 300 mm. Their surfaces were turned prior to URCP at a cutting speed of 180 m/min, a cutting depth of 0.2 mm, and a feed rate of 0.05 mm/r, with an initial surface hardness of 50 HRC (Rockwell) and an average surface roughness of 1.138  μm. URCP tests were conducted on the rods using the same processing parameters as those applied in the simulation. Each processed section was 3 mm in length, with a 1 mm gap between adjacent test sections to apply different processing parameters.

### 3.2 Methods

**Fig. 17:**
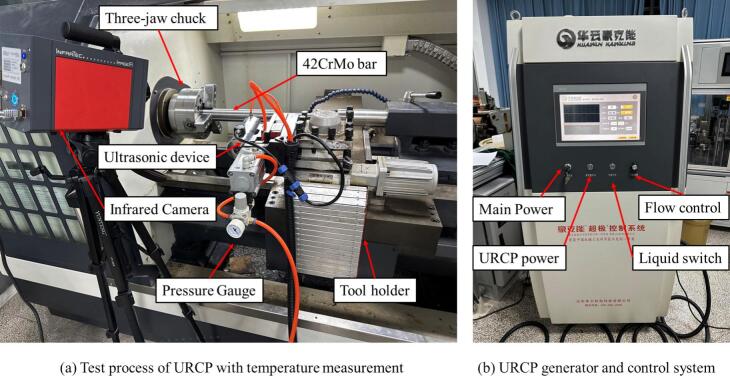
URCP strengthening system equipped with infrared thermographic monitoring.

During testing, the 42CrMo steel rod—after quenching, tempering, and turning—was clamped using a three-jaw hydraulic chuck on a ZAK4085D1 CNC lathe. The URCP tool ((trademark: HUAWIN, manufactured by Shandong Huayun Haokeneng) was mounted on the turret of the CNC lathe, and the ultrasonic rolling process is illustrated in Fig. 17. An INFRATEC infrared thermal imager with a resolution of 0.1 °C was employed to monitor the temperature evolution during the URCP. The CNC programming for the URCP was carried out via the FANUC system integrated into the CNC lathe. The end of the URCP device was connected to an air compressor via a pressure gauge, as shown in Fig. 17a. Two orange tubes were used: one was a pneumatic pressure line, which regulated the static pressure applied in URCP through adjustments to the dial on the pressure gauge; the other was a cooling fluid supply line, which delivered fully synthetic lubricant to cool the workpiece surface during processing, thereby reducing temperature rise. Ultrasonic amplitude and frequency were controlled via a generator unit connected to the URCP device, as shown in Fig. 17b. This generator also controlled the flow rate and switch of the lubricating coolant. The ultrasonic rolling equipment is designed with a total rated power of 2 kW, while the actual output power delivered to the rolling head is approximately 520 W.

Post-URCP, surface hardness was measured using an SF150 Rockwell hardness tester, manufactured by Mitutoyo Changzhou, China. Residual compressive stress was evaluated using the Xstress3000 portable stress measurement system, manufactured by Stresstech, Finland. For residual stress, hardness, and surface roughness analysis, the treated 42CrMo steel rods were sectioned using an electric discharge wire-cutting machine based on the different test zones. Samples were cut into cubes of 8  mm × 8 mm × 8 mm and then polished using a grinding and polishing machine. Sequential grinding was performed with waterproof silicon carbide papers of 500, 800, 1000, 1500, 2000, 2500, and 3000 grits for rough and semi-fine polishing. Final fine polishing was performed using W3.0 diamond paste. Throughout the polishing process, clean water was used for lubrication and cooling to avoid the introduction of additional residual stresses. All specimens underwent the same pre-treatment procedure prior to hardness and residual stress testing. Residual stress was analyzed using the sin^2^ψ method with a copper target. A total of 13 tilt angles (ψ) ranging from 0° to ±60° were employed to ensure the reliability and accuracy of the linear fitting. This range of tilt angles allows for robust determination of residual stress components based on the slope of the linear relationship between the measured lattice spacing and sin^2^ψ, with testing voltage and current set to the default values of 30 kV and 7 mA, respectively. Three points were measured in each test region, and the average was calculated to reduce experimental error. Hardness testing was also performed at three different positions along the same surface layer in each processed section, and the average value was taken to improve accuracy. Surface roughness was measured and analyzed using a MarSurf M280 surface profilometer over a measurement length of 4 mm, with a cut-off length (λc) of 0.8 mm. Each test section was measured three times, and the average value was taken to enhance precision and repeatability.

### 3.3 Experimental results of URCP

**Fig. 18:**
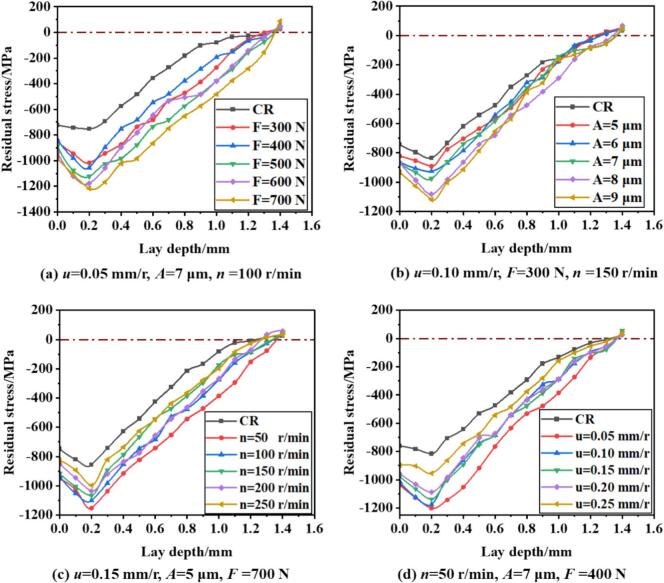
Depth-wise distribution of residual stress under URCP and CR.

To validate the accuracy of the URCP simulation model established in the previous section, URCP and CR experiments were conducted using identical processing parameters. As shown in Fig. 18a, the distribution of residual stress along the depth of the surface layer under different static pressures is presented. Similar to the simulation results, the maximum compressive residual stress increases with increasing static pressure in the URCP process. Specifically, when the static pressure reaches 700 N, the maximum compressive residual stress is −1217 MPa, whereas at 300 N it is only −1017 MPa. As the pressure increases from 400 N to 600 N, the stress increases incrementally by 38 MPa, 107 MPa, and 161 MPa, respectively. This is because higher static pressure enhances the plastic deformation capacity of the surface material, resulting in greater compressive residual stress. In contrast, under conventional rolling without ultrasonic excitation, the maximum residual compressive stress reaches only −752 MPa. This clearly demonstrates that URCP significantly outperforms CR in inducing residual compressive stress. Although there are differences between the absolute values obtained from simulation and experiment, the overall trend of residual stress variation with depth is consistent with the simulation results presented in Section 2. The maximum relative error remains within 10 %, confirming that the URCP simulation model has high accuracy and can effectively predict the ultrasonic rolling process.

Fig. 18b illustrates the influence of different ultrasonic amplitudes on the residual stress distribution along the depth. As the amplitude increases, the maximum residual compressive stress in the URCP experiment also increases. At an amplitude of 5 μm, the maximum stress is −892 MPa. With each 1 μm increment in amplitude, the maximum stress increases to −926 MPa (6 μm), −977 MPa (7 μm), and −1083 MPa (8 μm). When the amplitude reaches the upper limit of 9 μm, the maximum residual compressive stress peaks at −1120 MPa. This significant increase beyond 7 μm suggests that the system reaches an optimal excitation frequency, where ultrasonic energy most effectively promotes plastic deformation, thus enhancing the residual stress. This trend closely matches the simulation results in Section 2, further validating the model. For comparison, under CR conditions without ultrasonic assistance, the maximum residual stress is only −835 MPa. Therefore, the experimentally observed trend of residual stress variation with amplitude is consistent with the simulation predictions, reinforcing the accuracy of the URCP model.

Fig. 18c presents the residual stress distribution under different workpiece rotational speeds for both URCP and CR. Without ultrasonic excitation, the CR process produces a maximum residual stress of −856 MPa. In contrast, under URCP conditions with a rotational speed of 250 r/min, the maximum stress increases to −997 MPa. As the speed decreases to 200 r/min, 150 r/min, and 100 r/min, the stress further rises to −1035 MPa, −1067 MPa, and −1100 MPa, respectively. Although the increase in stress is gradual within this speed range, a significant jump is observed when the speed drops to 50 r/min, with residual stress reaching −1153 MPa. This sharper increase at low speeds is due to prolonged contact time between the rolling ball and the workpiece surface, enhancing the coupling effect between static pressure and ultrasonic excitation, thus intensifying plastic deformation and increasing energy transfer efficiency. This observation is in line with the simulation trends discussed in Section 2, further confirming the reliability of the simulation model.

Fig. 18d shows the effect of feed rate on residual stress under CR and URCP conditions. Without ultrasonic assistance, CR at a feed rate of 0.25 mm/r yields a maximum residual stress of −816 MPa at a depth of 0.2 mm. When ultrasonic vibration is applied, this value increases to −954 MPa under the same feed rate, demonstrating the significant enhancement effect of URCP over CR. As the feed rate decreases, the maximum residual stress continues to rise. At 0.20 mm/r, the stress reaches −1087 MPa. However, further reduction in feed rate results in a slower increase in stress. At 0.15 mm/r and 0.10 mm/r, the stress increases by only 51 MPa and 88 MPa, respectively. The most significant increase occurs when the feed rate is reduced from 0.25 mm/r to 0.20 mm/r. When the feed rate is further reduced to 0.05 mm/r, the maximum residual stress peaks at −1201 MPa. This trend is attributed to the longer contact time between the rolling ball and the workpiece at low feed rates, allowing sufficient ultrasonic energy to be transferred to the surface, thereby promoting grain refinement, dislocation density increase, and enhanced plastic deformation. However, once the material reaches its yield limit, further improvements in residual stress become negligible, indicating saturation of the strengthening effect. Overall, the experimental findings on feed rate effects are consistent with the simulation results in Section 2. Despite minor differences in stress values at various depths, the overall trends and magnitudes of variation agree well with the simulations. With the maximum relative error remaining within 10 %, it can be concluded that the URCP simulation model is both accurate and effective in representing the actual ultrasonic rolling process.

In summary, although discrepancies exist between simulated and experimental URCP results, the trends are highly consistent. The maximum relative error is within 10 %, which can be attributed to differences in mesh refinement and material properties. This confirms the robustness and predictive accuracy of the established URCP simulation model.

**Fig. 19:**
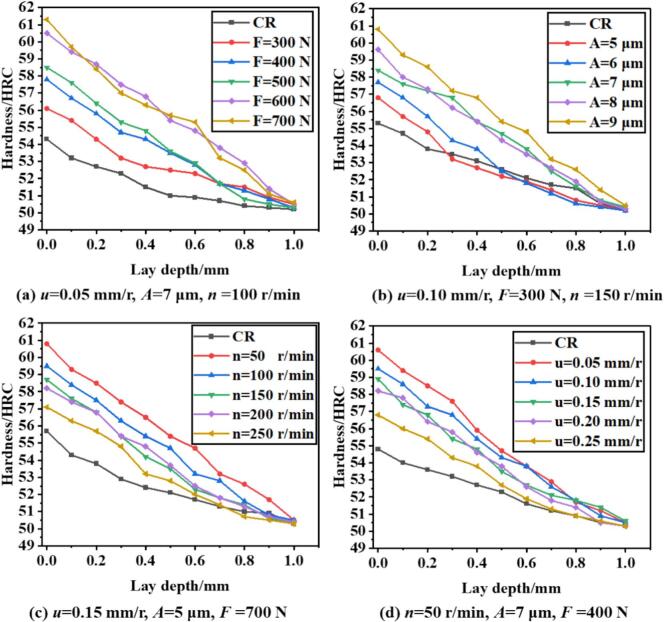
Variation in surface hardness of 42CrMo steel under URCP and CR.

To further illustrate the comparative results between the URCP and CR observed in both the experimental tests and the simulations discussed in Section 2, hardness measurements were conducted at varying subsurface depths. To minimize measurement errors, the hardness at each depth was averaged from three different points. Fig. 19a shows the variation of hardness with depth under different processing conditions. In the absence of ultrasonic vibration, conventional rolling increased the surface hardness of 42CrMo steel from an initial value of 50 HRC to 54.3 HRC. When ultrasonic energy was applied, the surface hardness increased rapidly to approximately 56 HRC under a static pressure of 300 N. With further increases in static pressure, the surface hardness continued to rise monotonically. Specifically, at static pressures of 400 N, 500 N, and 600 N, the surface hardness reached 57.8 HRC, 58.5 HRC, and 60.5 HRC, respectively. When the static pressure was increased to 700 N, the surface hardness peaked at 61.3 HRC — the maximum value under the tested global parameter combinations. The trends observed in the URCP hardness measurements were consistent with those predicted by the simulations in the previous section. The maximum relative error between experimental and simulation results was controlled within 10 %, validating the accuracy of the URCP simulation model.

Fig. 19b presents the hardness distribution with depth under different ultrasonic vibration amplitudes. At an amplitude of 5 μm, the surface hardness reached 56.8 HRC. As the amplitude increased, the surface hardness continued to rise. When the amplitude reached 6 μm, the hardness increased to 57.7 HRC, and at 9 μm, the maximum value of 60.8 HRC was recorded. Hardness gradually decreased with increasing depth, returning to its original (untreated) level at approximately 1 mm beneath the surface. In contrast, under CR conditions without ultrasonic vibration, the surface hardness only increased to 55.3 HRC. These results demonstrate that the experimental trend of increasing hardness with vibration amplitude is consistent with the simulation results. The simulated surface hardness at an amplitude of 9 μm was 60.5 HRC, while at 5 μm it was 56.6 HRC. The differences between the experimental and simulated values were small, indicating good agreement and model accuracy. However, these discrepancies could be attributed to variations in ultrasonic coupling efficiency during the actual processing. In simulations, ultrasonic energy is assumed to be a stable and continuous input. In practice, however, the energy transfer between the ultrasonic vibration and the workpiece is influenced by factors such as static pressure and lubrication, which can lead to fluctuations and affect the actual plastic deformation and strengthening effects. Overall, the URCP model successfully captures the primary mechanisms behind surface hardness enhancement and demonstrates strong predictive capability.

Fig. 19c illustrates the influence of workpiece rotational speed on the hardness distribution with depth. Without ultrasonic assistance, CR processing increased the surface hardness only to 55.7 HRC. In contrast, URCP significantly enhanced the surface hardness. Notably, at a rotational speed of 50 r/min — the lowest among the test conditions — the surface hardness reached 60.8 HRC. Even at higher speeds, the surface hardness remained as high as 57.1 HRC, which was still significantly greater than that achieved with CR alone. The experimental trend of decreasing hardness with increasing rotational speed was consistent with the simulation predictions. The decline in surface hardness at higher speeds can be explained by reduced contact time between the rolling ball and the workpiece surface. It is also important to note that surface hardness in the simulations was not directly extracted from ABAQUS post-processing but rather estimated from equivalent plastic strain using an empirical relationship for 42CrMo steel. The simulation assumed an idealized rolling contact model, leading to a more concentrated stress distribution and potentially overestimated hardening effects. In contrast, actual Rockwell hardness measurements are affected by surface quality and indentation location. Despite these differences in absolute values, the experimental and simulation trends remain highly consistent, further validating the reliability of the simulation model.

Fig. 19d presents the evolution of surface hardness under different feed rates during URCP. As the feed rate decreased, surface hardness generally increased. At a feed rate of 0.25 mm/r, the surface hardness reached 56.8 HRC. In comparison, under CR conditions without ultrasonic vibration, the surface hardness was only 54.8 HRC. As the feed rate decreased incrementally by 0.05 mm/r, the surface hardness increased to 58.2 HRC at 0.20 mm/r, 58.9 HRC at 0.15 mm/r, and 59.5 HRC at 0.10 mm/r. A further reduction to 0.05 mm/r resulted in the highest surface hardness of 60.6 HRC. Notably, the hardness increase from 0.25 mm/r to 0.20 mm/r was significantly greater than the increase from 0.20 mm/r to 0.10 mm/r. This observation aligns with the simulation results from the previous section. When the feed rate is lower than 0.25 mm/r, the contact time between the rolling tool and workpiece surface increases, allowing for more intense plastic deformation and vibrational impact on each surface point. This leads to severe lattice distortion, dislocation accumulation, and grain refinement near the surface — all of which contribute to hardness enhancement. However, when the feed rate is between 0.10 mm/r and 0.15 mm/r, the strengthening effect begins to saturate. At these low feed rates, the surface has already undergone substantial impact and deformation, resulting in diminishing returns in hardness improvement. Although there were differences between the experimental and simulated hardness values, the overall trend was highly consistent, reaffirming the accuracy and reliability of the URCP simulation model.

**Fig. 20:**
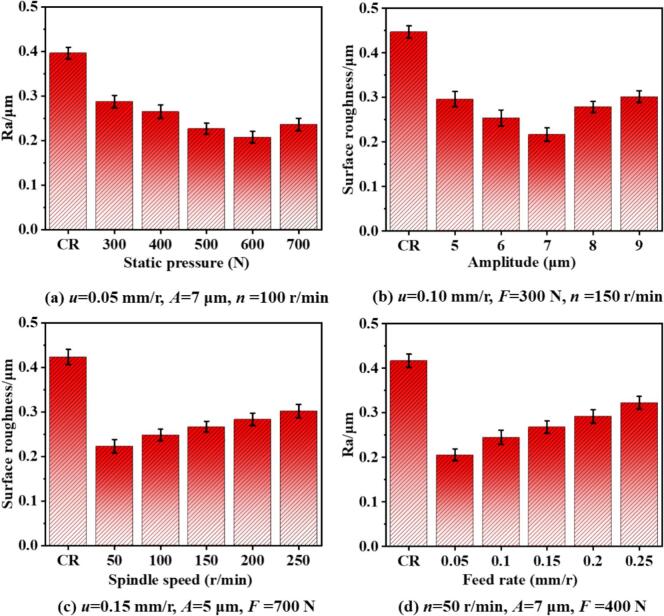
Ra variation under URCP and CR.

Fig. 20 presents the variations in surface roughness under identical processing parameters, serving to validate the accuracy of the URCP simulation model described in the previous section.

In Fig. 20a, the changes in surface roughness under different static pressures during URCP and CR are shown. Without ultrasonic energy input, the surface roughness after CR reaches 0.396 μm. However, once high-frequency ultrasonic vibration is introduced, the surface roughness of 42CrMo steel is significantly reduced. When the static pressure is 300 N, the surface roughness drops to 0.287 μm. As the pressure increases to 400 N, it further decreases to 0.265 μm. At 500 N, the roughness is 0.227 μm, and reaches its global minimum of 0.208 μm at 600 N. However, increasing the pressure to 700 N results in a slight increase to 0.236 μm. This indicates a trend of first decreasing and then increasing surface roughness as static pressure increases from 300 N to 700 N, with the lowest value of 0.208 μm at 600 N. This trend is in high agreement with the simulation results, which also predict a minimum roughness of 0.217 μm at 600 N. At this pressure, the coupling between ultrasonic frequency and static pressure enhances the stability of the ultrasonic system, leading to more uniform and stable high-frequency impacts from the rolling ball and thus a smoother surface. The small relative error between experimental and simulation values further supports the accuracy of the established URCP simulation model.

Similarly, Fig. 20b shows the effect of ultrasonic amplitude on surface roughness in URCP and CR processes. The influence of amplitude on surface roughness follows a trend similar to that of static pressure. As the amplitude increases from 5 μm to 9 μm, the surface roughness initially decreases and then increases. The minimum value of 0.217 μm is achieved at an amplitude of 7 μm. At 6 μm and 8 μm, the roughness values are 0.254 μm and 0.279 μm, respectively. In contrast, the surface roughness under CR without ultrasonic assistance reaches 0.447 μm—substantially higher than all URCP cases. Both excessively small and large amplitudes can destabilize the ultrasonic system, causing surface rebound or accumulation phenomena. These effects disrupt continuous processing, reduce effective contact time, and ultimately impair surface quality. The experimental trends closely mirror those observed in the simulation results, further confirming the coherence and precision of the simulation model despite numerical differences under specific parameters.

Fig. 20c illustrates the influence of workpiece rotational speed on surface roughness in URCP and CR processes. In CR, the surface roughness is 0.447 μm. Under ultrasonic assistance, however, roughness is significantly reduced. The lowest surface roughness of 0.223 μm is achieved at 50 r/min. As the rotational speed increases to 100, 150, 200, and 250 r/min, the surface roughness correspondingly rises to 0.248 μm, 0.267 μm, 0.283 μm, and 0.302 μm, respectively. Both simulation and experimental results show that higher rotational speeds may cause the rolling ball to bounce, leaving unprocessed areas on the surface, thereby increasing roughness. Conversely, lower speeds ensure prolonged and stable contact between the ball and the workpiece, enhancing the surface finishing effect. The consistent monotonic increase in roughness with speed, along with minimal deviation between simulation and experimental data, confirms the robustness and reliability of the URCP simulation model.

Lastly, Fig. 20d shows the effect of different feed rates on surface roughness in URCP. The minimum surface roughness of 0.206 μm is observed at a feed rate of 0.05 mm/r. As the feed rate increases to 0.10, 0.15, 0.20, and 0.25 mm/r, the surface roughness increases to 0.245 μm, 0.268 μm, 0.292 μm, and 0.323 μm, respectively. Nevertheless, all these values remain significantly lower than that under CR (0.417 μm). The influence of feed rate on surface roughness is analogous to that of rotational speed, as both affect the contact time between the rolling ball and the workpiece. At lower feed rates, the ball has more time to interact with the surface, allowing ultrasonic energy to be fully transferred and yielding more uniform plastic deformation. This results in a smoother and higher-quality surface. Hence, URCP proves superior to conventional rolling in significantly improving the initial surface quality of the material. The alignment of trends between simulation and experimental outcomes under varying feed rates further validates the accuracy of the developed simulation model.

**Fig. 21:**
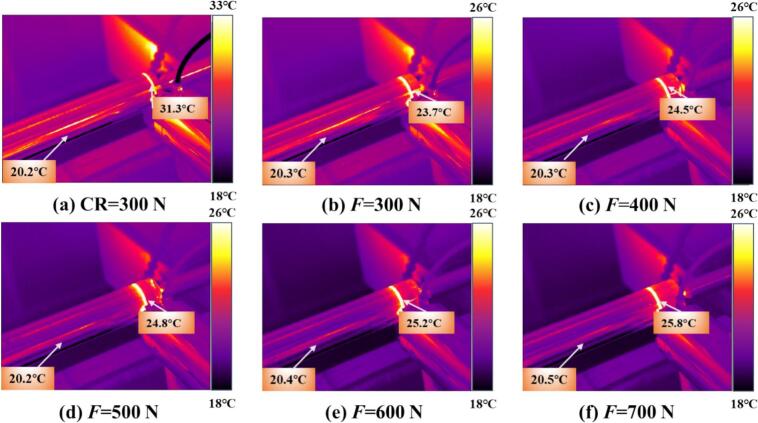
Measured surface temperature of CR and URCP under different static pressures (*u* = 0.05 mm/r, *A* = 7 μm, *n* = 100 r/min).

To further validate the simulation model of thermomechanical coupling for 42CrMo steel established in the previous section, temperature measurements were conducted during the URCP. As shown in Fig. 21, the surface temperature variation of 42CrMo steel under different static pressures is illustrated. Initially, the surface temperature of the 42CrMo steel bar is the same as the ambient room temperature, approximately 20.2 °C. During CR, a high-temperature contact zone is formed between the rolling ball and the workpiece, with the temperature in the contact region reaching up to 31.3 °C. In contrast, during the URCP process, the temperature in the contact area remains significantly lower, well below 30 °C. As the static pressure increases, the temperature in the processing zone gradually rises. When the static pressure is set at 300  N, the contact temperature is only 23.7 °C. As the pressure is further increased, the surface temperature of the 42CrMo bar fluctuates slightly around 20 °C, while the contact area experiences a slight rise. At 400  N, the processing temperature rises to 24.5 °C. When the pressure is increased to 500  N and 600  N, the surface temperature increases by 0.9 °C and 1.5 °C, respectively, compared to that at 300  N. At 700  N, the temperature rises to 25.8 °C.

Overall, the surface temperature in the URCP region increases with static pressure; however, the absolute temperature rise remains significantly lower than that in conventional rolling. This demonstrates that URCP can effectively reduce the surface temperature in the processing zone. Moreover, this trend is consistent with the simulation results presented in the previous section. Although discrepancies exist between the absolute temperature values from experiments and simulations—mainly due to infrared thermometer measurement errors—the overall trends align. Infrared thermometers capture peak local temperatures, whereas the simulation outputs average equivalent temperatures. Additionally, the simulation did not account for heat dissipation in the actual environment, which can result in measured temperatures occasionally being higher than simulated ones. Despite simplifications in the URCP simulation model leading to some differences, the overall trend consistency confirms the model’s applicability for subsequent process parameter optimization.

**Fig. 22:**
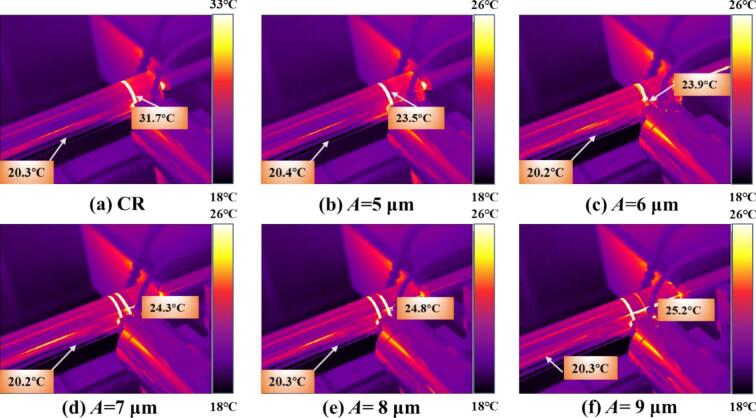
Measured surface temperature of CR and URCP under different amplitudes (*u* = 0.10 mm/r, *F* = 300 N, *n* = 150 r/min).

As shown in Fig. 22, the variation of absolute surface temperature rise under different ultrasonic amplitudes is presented. Similarly, under a room temperature environment of around 20 °C, the surface temperature of 42CrMo steel fluctuates near 20.4 °C. During conventional rolling without ultrasonic vibration, the surface temperature of the material rapidly increases to 31.7 °C—a rise of more than 10 °C. However, under URCP with an ultrasonic amplitude of 5  μm, the surface temperature increases by only 3.1 °C, significantly lower than that in CR. As the ultrasonic amplitude increases, the surface temperature also rises. At 6  μm, the temperature at the contact area increases to 23.9 °C; at 7  μm, it rises to 24.3 °C; and when the amplitude reaches 9  μm, the absolute surface temperature rise reaches 5.1 °C, with the local temperature in the contact region reaching 25.2 °C. At 8  μm, the surface temperature falls between those at 7  μm and 9  μm.

These results confirm that URCP can significantly reduce the absolute temperature rise during the strengthening process, with surface temperatures much lower than those in conventional rolling. Moreover, the absolute temperature rise increases steadily with ultrasonic amplitude, consistent with the simulation results in the previous section. This consistency highlights the strong predictive capability of the URCP simulation model for temperature evolution. Although the absolute temperature rise measured in experiments is slightly higher than that in simulations—due to simulations yielding steady-state average temperatures and infrared measurements capturing local peak temperatures—the overall agreement in trends further validates the feasibility and effectiveness of the simulation model in analyzing temperature evolution behavior.

**Fig. 23:**
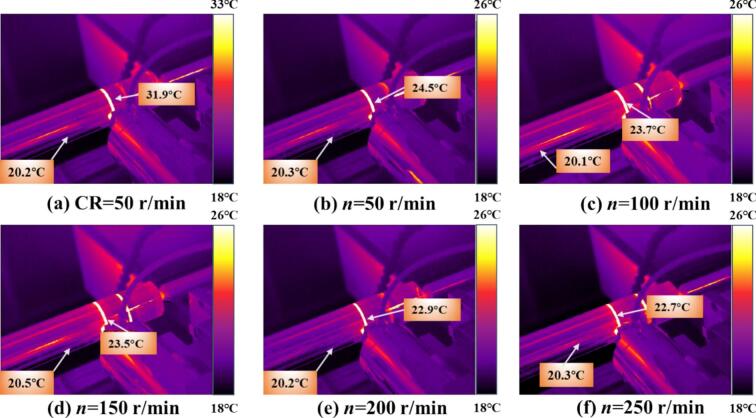
Real-time surface temperature of CR and URCP at different spindle speeds (*u* = 0.15 mm/r, *A* = 5 μm, *F* = 700 N).

As shown in Fig. 23, the infrared thermometer measurements reveal the variation in processing temperature in the contact region under different workpiece rotation speeds. It can be observed that as the rotational speed of the workpiece increases, the temperature at the contact area between the rolling ball and the workpiece during URCP decreases. When the workpiece rotates at 50 r/min, the annular contact region formed between the rolling ball and the workpiece reaches a temperature of 24.5 °C. As the rotation speed increases to 100 r/min, the temperature in the URCP contact region drops to 23.7 °C. With further increases to 150 r/min and 200 r/min, the surface temperatures continue to decrease to 23.5 °C and 22.9 °C, respectively. At the maximum speed of 250 r/min, the surface temperature in the contact area drops to the lowest recorded value of 22.7 °C during the entire URCP. In contrast, for CR, the surface temperature in the contact strengthening area of the 42CrMo steel rod reaches 31.9 °C—significantly higher than that observed in URCP. This confirms that URCP is effective in significantly reducing the absolute temperature rise during the strengthening process of 42CrMo steel. Moreover, with increasing workpiece speed, the contact time per unit area between the rolling ball and the workpiece shortens due to the faster circumferential motion. This enhances heat dissipation and prevents heat buildup in localized regions. In contrast, lower rotational speeds lead to more severe plastic deformation and longer contact time, intensifying frictional heat generation. Although ultrasonic assistance is present, the URCP’s separation and cooling effects are not fully activated under fixed ultrasonic frequency at low speeds. The observed trend of decreasing temperature with increasing workpiece speed is consistent with the simulation results presented in the previous section. The relatively small discrepancy between the experimental and simulated absolute temperature rise further validates the accuracy of the URCP thermal–mechanical coupling simulation model.

**Fig. 24:**
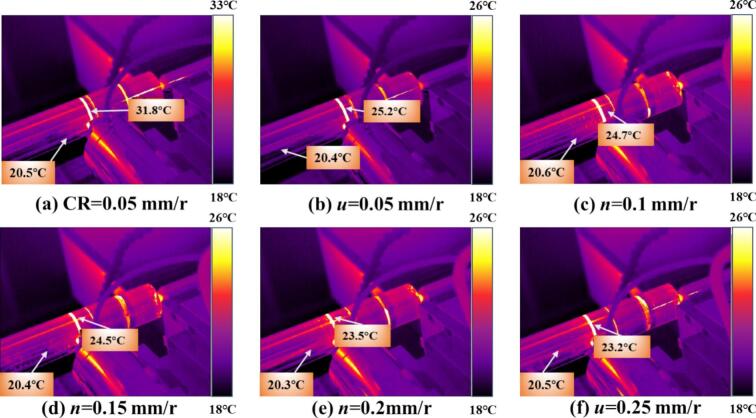
Measured surface temperature of URCP at different feed rates (*n* = 50 r/min, *A* = 7 μm, *F* = 400 N).

As shown in Fig. 24, the variation of absolute temperature rise in 42CrMo steel under different feed rates is illustrated. When the feed rate is 0.05 mm/r, the surface temperature in the rolling contact area reaches 25.2 °C, while the non-strengthened area remains around 20.4 °C. As the feed rate increases, the processing temperature in the contact region decreases to 24.7 °C, 24.5 °C, and 23.5 °C. At a feed rate of 0.25 mm/r, the surface temperature drops to the lowest value of 23.2 °C. This temperature reduction can be attributed to the shortened contact time between the rolling ball and the workpiece at higher feed rates, which reduces frictional heating. The generated heat cannot accumulate effectively due to the insufficient time for the surface to warm up. Additionally, faster feed speeds cause the rolling ball to travel a greater distance per unit time, resulting in shorter and quicker contact with each point on the workpiece. Under such conditions, ultrasonic vibrations—operating in a continuous hammering mode—are more effective at dissipating heat generated during processing, further reducing friction-induced temperature rise. High-speed feed enables the rolling ball to remove more heat, and the broader heat diffusion range reduces the extent of thermal stress, ultimately lowering the surface temperature peaks. Compared to conventional rolling, the absolute temperature rise in URCP remains significantly lower. Under CR conditions, the surface temperature increases to 31.8 °C. The observed experimental trends—particularly the decrease in temperature with increasing feed rate—align well with the simulation results presented in the previous section. Therefore, it can be concluded that the developed thermal–mechanical coupled simulation model for ultrasonic rolling of 42CrMo steel is highly accurate and reliable, with strong predictive capability for temperature evolution and effectiveness in optimizing subsequent URCP parameters.

## 4 URCP surface performance multi-objective optimization

### 4.1 Objective functions and optimization parameters

According to the simulation results presented in the previous section, the thermomechanical coupling effect of the URCP significantly enhances the surface mechanical properties of the material by increasing the residual compressive stress and equivalent plastic strain. However, to further improve these surface properties, a multi-objective optimization algorithm is required to fine-tune the process parameter domain. This approach helps resolve conflicts among various surface performance indicators and mitigates the mismatch between process parameters and surface quality. For example, while increasing static pressure and vibration amplitude can enhance residual compressive stress, excessive values may lead to surface cracking or even failure, thereby increasing surface roughness. Therefore, to maximize the benefits of ultrasonic rolling in terms of residual compressive stress, hardness, and surface quality, a multi-objective optimization of URCP process parameters is necessary. The optimization objectives are derived from simulation data. A quadratic response surface methodology (RSM) is employed to establish the multi-objective optimization model for URCP surface performance. This method offers high prediction accuracy and captures the interactions between different process parameters, includes quadratic terms of the parameters, and accounts for the error between predicted and actual values. The general form of the quadratic RSM model is shown in Eq. (8). An analysis of variance (ANOVA) confirms that the prediction model has good consistency with experimental data. The optimization parameters include spindle speed, feed rate, static pressure, and ultrasonic amplitude. The optimization objectives focus on minimizing surface roughness while maximizing residual compressive stress and hardness.

(8) $$M_{p}=S_{0}+\sum_{i=1}^{n}S_{i}\gamma_{i}+\sum_{i=1}^{n}\sum_{j=1,i<j}^{n}S_{\mathrm{ij}}\gamma_{i}\gamma_{j}\sum_{i=1}^{n}S_{\mathrm{ii}}\gamma_{i}^{2}+\upsilon$$

In Eq. (8), $S_{0}$ is the intercept term in the response surface model; $S_{i}$ represents the linear coefficients of URCP process parameters; $S_{\mathrm{ij}}$ denotes the quadratic coefficients of URCP process parameters; υ represents the error between the actual surface performance and the predicted model values; $\gamma_{i}$ and $\gamma_{j}$ represent combinations of different process parameters in URCP; $\gamma_{i}^{2}$ denotes the square of the rolling process parameter values in URCP.

Based on the simulation results summarized in Table 4, 25 sets of orthogonal test data for process parameters and surface properties were substituted into the RSM model, as shown in Eq. (8). After excluding abnormal data points, the following URCP multi-objective optimization models were established, as shown in Eqs. (9), (10), (11).

(9) $$\begin{array}{c} \mathrm{RCS}=886+2.444n-35.2A+0.542F+479u+0.003925n^{2}\\ +5.78A^{2}-0.000430F^{2}+2260u^{2}-0.0752nA-\\ 0.000541nF-2.011nu-129.4Au \end{array}$$

(10) $$\begin{array}{c} \mathrm{HRC}=55.235+0.00807n+1.321A-0.00816F+16.67u-0.000030n^{2}\\ -0.07779A^{2}+0.000008F^{2}-17.42u^{2}+0.000756nA+\\ 0.000006nF-0.04942nu-0.507Au \end{array}$$

(11) $$\begin{array}{c} \mathrm{Ra}=0.1464+0.000779n+0.0661A-0.000403F-1.702u-0.000004n^{2}\\ -0.00553A^{2}+0.000001F^{2}+1.736u^{2}-0.000013nA+\\ 0.000001nF+0.001034nu+0.1365Au \end{array}$$

In these equations, RCS represents the predicted residual compressive stress in URCP; HRC denotes the predicted hardness; Ra refers to the predicted surface roughness after URCP. n represents the spindle speed; F is the static pressure; u is the feed rate; A represents the ultrasonic amplitude.

**Fig. 25:**
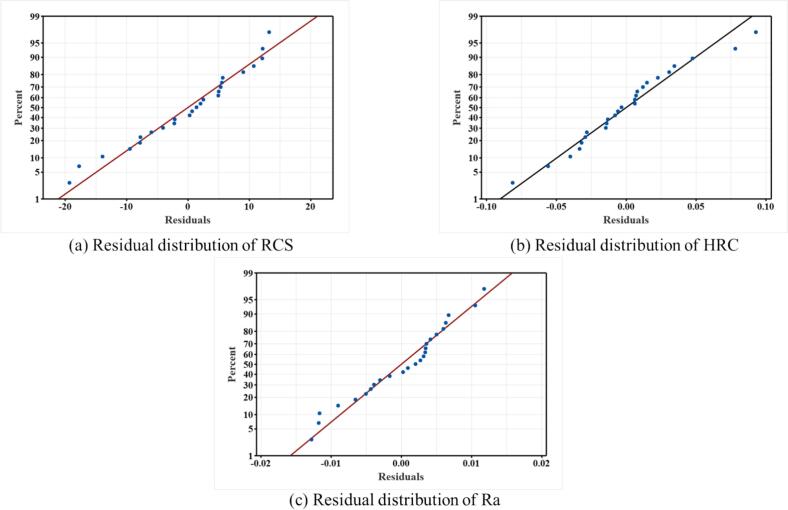
Residual distribution of surface properties after URCP.

Fig. 25 presents the residual distribution of the surface performance model in URCP, showing that the sample points are evenly distributed on both sides of the baseline, indicating good uniformity. According to the ANOVA results in Table 5 and F-test values, the model shows high accuracy, with a p-value less than 0.05 and F-values greater than 1. This indicates that the prediction model has over 95 % confidence, showing good reliability and fitting, and can be effectively used for predicting surface performance and optimizing URCP process parameters.

**Table 5:** Analysis of variance (ANOVA) of the URCP predictive model for 42CrMo steel.

Surface property	degrees of freedom	predicted sum of squares	predicted mean square	F-value	Predicted *R^2^*
RCS	12	51178.4	4264.9	25.99	96.30 %
HRC	12	7.45367	0.621139	207.45	97.61 %
Ra	12	0.029754	0.002479	26.97	96.43 %

Thus, the goal of optimizing URCP surface performance is formulated as the objective function shown in Eq. (12). The optimization aims to minimize surface roughness and maximize hardness and residual compressive stress. Since residual compressive stress is a negative value, multiplying it by a negative sign effectively converts it into a minimization problem.

(12) $$\mathrm{Objective}=\left\{\begin{array}{c} -\text{Min}\;f_{\mathrm{RCS}}\left(F,A,n,u\right)\\ \text{Max}\;f_{\mathrm{HRC}}\left(F,A,n,u\right)\\ \text{Min}f_{\mathrm{Ra}}\left(F,A,n,u\right) \end{array}\right)$$

(13) $$\mathrm{Parameterrange}=\left\{\begin{array}{c} 300N⩽F⩽700N\\ 6\mu m⩽A⩽9\mu m\\ 50r/\min⩽n⩽250r/\min\\ 0.05mm/r⩽u⩽0.20mm/r \end{array}\right)$$

The optimization parameter ranges are set based on the 25 orthogonal experiments listed in Table 4. Specifically: The spindle speed is set between 50r/min and 250r/min; Ultrasonic amplitude is set between 6 μm and 9 μm; Static pressure is adjusted within 300N to 700N based on simulation results and the limits of the CNC lathe; The feed rate is set between 0.05mm/r and 0.20mm/r. The specific range of URCP process parameters for optimization is shown in Eq. (13).

### 4.2 PSSAO algorithm optimization process

In previous optimization studies of ultrasonic rolling parameters, conventional algorithms such as Simulated Annealing (SA) and Particle Swarm Optimization (PSO) [41] were applied to determine the optimal parameter domain for ultrasonic rolling. While these traditional multi-objective optimization algorithms—originating from classical genetic algorithms—are effective within certain application domains, they still suffer from several limitations. These include low optimization efficiency due to excessive iterations and an inability to produce a well-distributed Pareto front when handling complex conflicts between surface properties. To address the inefficiencies and local optima issues commonly encountered with single traditional genetic algorithms in solving complex nonlinear multi-objective problems, this study proposes a novel improved optimization algorithm: the Particle Swarm Simulated Annealing Optimization (PSSAO) algorithm.

**Fig. 26:**
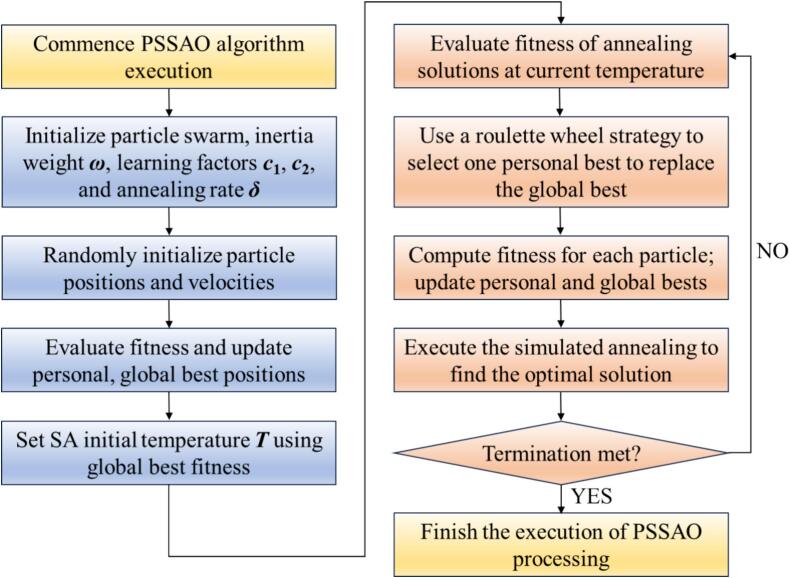
Principle diagram of the PSSAO optimization algorithm.

By integrating SA and PSO, the method compensates for the shortcomings of each individual algorithm and enhances their complementarity. This hybrid strategy is designed to tackle the intricate trade-offs among surface properties in URCP, enabling the identification of optimal processing parameters that yield superior surface performance for 42CrMo steel. In the context of SA and PSO, the core advantage of simulated annealing lies in its ability to escape local optima by setting a high initial temperature and decreasing it slowly, potentially allowing for global optimum discovery. However, this slow cooling rate and elevated initial temperature also lead to inefficient optimization, often requiring thousands of iterations. Moreover, the use of the Metropolis criterion to determine the acceptance probability of a new solution introduces randomness and uncertainty, making the optimization outcome less predictable. On the other hand, PSO features a much faster optimization rate and can converge to optimal solutions with relatively few iterations. However, this rapid convergence often causes premature entrapment in local optima due to insufficient global exploration, particularly in the early stages. In later iterations, the convergence speed declines, and its global search capability becomes limited compared to SA. To overcome these limitations, the proposed PSSAO algorithm fuses the global exploration strength of SA with the rapid convergence of PSO. Specifically, the Metropolis criterion from SA is introduced into the global optimization process of PSO to allow particles to escape local optima probabilistically. This hybrid method not only accelerates the optimization process compared to SA alone but also mitigates PSO’s tendency to prematurely converge, thus achieving a more efficient, globally oriented, and accurate multi-objective optimization strategy. This is critical for rapidly identifying the optimal URCP process parameter domain. The detailed optimization procedure is illustrated in Fig. 26.

The PSSAO algorithm comprises two main components: particle swarm optimization and simulated annealing. The process begins by initializing a random population of particles. For each particle, its position and velocity are recorded, along with algorithm parameters such as inertia weight, learning factors, and the annealing rate used in SA. Using the initial positions and velocities, the fitness values of all particles are calculated based on the objective functions defined for the URCP multi-objective optimization. The current position and the historically best position for each particle are tracked, and the global best fitness value is identified. This optimal fitness value is then used to determine the initial temperature for the simulated annealing process, as shown in Eq. (14). Based on the current temperature and position, the SA mechanism is used to evaluate and update the optimal fitness value, further guiding the search away from local optima.

(14) $$f_{\mathrm{SA}}\left(l_{\mathrm{xd}}\right)=\frac{\exp\left(\frac{f\left(l_{\mathrm{xd}}\right)-f\left(l_{\mathrm{hb}}\right)}{T}\right)}{\sum {\;}_{x=1}^{n}\exp\left(\frac{f\left(l_{\mathrm{xd}}\right)-f\left(l_{\mathrm{hb}}\right)}{T}\right)}$$

The term $f_{\mathrm{SA}}\left(l_{\mathrm{xd}}\right)$ refers to the fitness value of the x−th particle in the d−th dimension, which corresponds to the objective functions in the ultrasonic rolling surface performance optimization process—namely, residual compressive stress $\left(\mathrm{RCS}\right)$, surface roughness $\left(\mathrm{Ra}\right)$, and surface hardness $\left(\mathrm{HRC}\right)$. The variable $l_{\mathrm{xd}}$ denotes the position of the x−th particle in the d−th dimension, which represents the ultrasonic rolling process parameters in the URCP multi-objective optimization, including spindle speed $\left(n\right)$, static pressure $\left(F\right)$, ultrasonic amplitude $\left(A\right)$, and feed rate $\left(u\right)$. The fitness value of this particle is represented by $f\left(l_{\mathrm{xd}}\right)$ of the x−th particle in the d−th dimension, while $f\left(l_{\mathrm{hb}}\right)$ indicates the global best position found so far across all particles. T is the initial temperature used in the simulated annealing process. To enhance global optimization capability, a roulette wheel selection mechanism is employed, where the optimal position of a randomly selected individual replaces the current global best position. In the PSSAO algorithm, all particles in the population are ranked based on their fitness values. Individuals with higher fitness have a greater probability of being selected, meaning the better a particle performs, the more likely it will be chosen by the algorithm. The selection procedure for determining the best individual is as follows. First, as shown in Eq. (15), a random number between 0 and 1 is generated. Then, the cumulative probability of each particle is calculated based on its fitness value under the simulated annealing framework in Eq. (16). The particle whose cumulative probability satisfies the condition defined in Eq. (17) is selected, and its position replaces the global best position for the next iteration.

(15) $$\tau =random\left(z\right)$$

(16) $$P\left(y\right)=\sum {\;}_{x=1}^{y}f_{\mathrm{SA}}\left(l_{\mathrm{xd}}\right)$$

(17) $$\tau \left(i-1\right)<\tau <\tau \left(i\right)$$

The particle velocity update rule in the PSSAO algorithm is given in Eq. (18):

(18) $$v_{\mathrm{xd}}^{a+1}=\omega_{1}v_{\mathrm{xd}}^{a}+c_{1}i_{1}\left(l_{\mathrm{xd}}^{a}-m_{\mathrm{xd}}^{a}\right)+c_{2}i_{2}\left(l_{\mathrm{hb}}^{a}-m_{\mathrm{hb}}^{a}\right)$$

Here, $v_{\mathrm{xd}}^{a+1}$ is the updated velocity of the x−th particle in the d−th dimension at generation a+1, $\omega_{1}$ is the inertia weight, $c_{1}$ and $c_{2}$ are the cognitive and social learning factors respectively, $i_{1}$ and $i_{2}$ are random numbers uniformly distributed in the range [0,1]. $v_{\mathrm{xd}}^{a}$ refers to the velocity of the particle at the same time before the update in the previous generation. $l_{\mathrm{xd}}^{a}$ indicates the historical best position of the particle at the x−th position, in the d−th dimension, in generation a during the entire optimization process. $m_{\mathrm{xd}}^{a}$ denotes the current position of the particle at the x−th position, in the d−th dimension, in generation a. $l_{\mathrm{hb}}^{a}$ refers to the historical best position of another particle in the d−th dimension, in generation a, which can be used to replace the global best position. $m_{\mathrm{hb}}^{a}$ denotes the current position of that other particle in generation a in the d−th dimension. Finally, as shown in Eq. (19), simulated annealing is applied to further refine the search. Whether the annealing process continues or stops depends on a predefined termination condition. If the condition is met, the search halts and the current parameters are taken as the optimal solution. If not, the algorithm returns to recalculate the particle fitness under the current temperature.

(19) T=δt

Where T is the current annealing temperature, δ is the annealing rate (a value less than 1), and t is the annealing time (iteration count). In this study, the parameter settings for the PSO part of the PSSAO algorithm are as follows: both learning factors $c_{1}$ and $c_{2}$ are set to 0.5, the population size is 30, and the inertia weight $\omega_{1}$ is 0.8. For the SA part, the initial temperature $T_{0}$ is set to 1, and the termination temperature is set to T 1 × 10^−6^. This setup governs the Metropolis criterion that determines the acceptance probability of new solutions. The proposed PSSAO algorithm is designed to accelerate the convergence of Pareto front optimization while addressing the limitations of conventional PSO and SA algorithms. By integrating the temperature decay and Metropolis acceptance criteria from SA into the PSO framework, the algorithm allows particles that are trapped in local optima to probabilistically accept inferior solutions, enabling them to escape and explore the global solution space more effectively. As a result, this hybrid algorithm facilitates fast, global, and accurate multi-objective optimization, achieving superior surface performance for 42CrMo steel in URCP processes and rapidly converging on the optimal processing parameter domain.

### 4.3 Optimization results

**Fig. 27:**
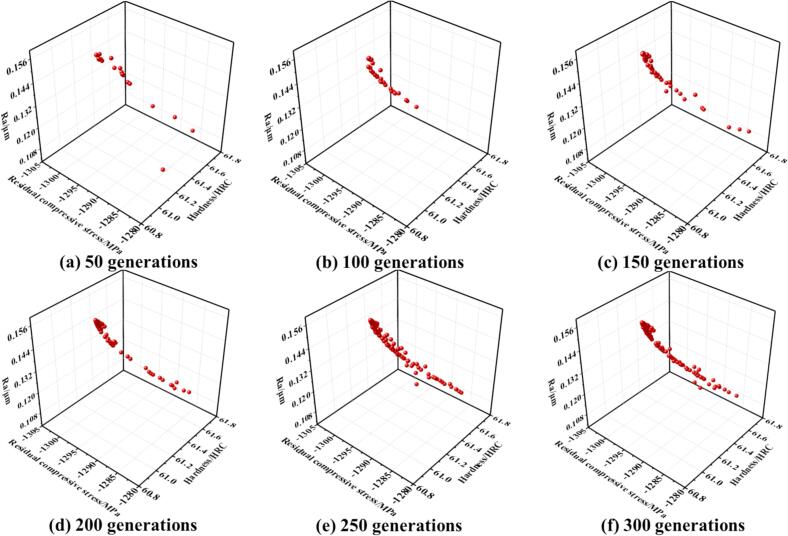
Pareto front of optimal solutions obtained by PSSAO at different iteration numbers.

Fig. 27 illustrates the evolutionary distribution of the Pareto-optimal solution sets in 3D space obtained by applying the proposed PSSAO algorithm to optimize the surface properties of ultrasonic rolling. These distributions correspond to different generations: 50, 100, 150, 200, 250, and 300. Based on the URCP multi-objective optimization mathematical model established in the previous section, the optimal surface performance and corresponding process parameters are analyzed. The optimization objectives include minimizing surface roughness (Ra), maximizing residual compressive stress (RCS), and maximizing hardness (HRC). Meanwhile, the process parameters to be optimized include workpiece rotational speed, static pressure, feed rate, and ultrasonic amplitude.

As shown in Fig. 27a, at the early stage of optimization (generation 50), the distribution of Pareto solutions is scattered and disorganized. This indicates that the optimal process parameter domain has not yet reached a desirable state, and the number of optimal solutions is still limited, suggesting that the population has not yet converged. At this stage, both the quality and diversity of individuals in the population remain low, and the optimization is still in a random exploration phase. As the number of iterations increases, Fig. 27b shows that by generation 100, the URCP solution set begins to gradually approach the Pareto front, with some solutions starting to cluster in the middle region. This suggests a slow convergence trend. By generation 150 (Fig. 27c), the Pareto-optimal solution set obtained by the PSSAO algorithm becomes noticeably more coherent and dense compared to generations 50 and 100. The fitness values of particles in the population continue to improve, with the distribution becoming increasingly concentrated in 3D space. The optimization performance of the PSSAO algorithm is significantly enhanced at this stage. As the number of iterations reaches 200 and then 250 (Figs. 27d and 27e), the Pareto-optimal solution sets for the surface properties under ultrasonic rolling become more uniformly and smoothly distributed. A clear curved surface is formed along the boundary, indicating that the PSSAO algorithm has reached a state of basic convergence and stability. At this point, a globally optimal and stable Pareto front is achieved. The algorithm successfully escapes local optima, and the quality and distribution of the solutions reach an ideal state. This allows for globally optimal performance, effectively resolving conflicts among surface properties in URCP and achieving their synergistic enhancement along with the optimal process parameter domain. As shown in Fig. 27f, when the number of iterations reaches 300, there is no significant improvement in the Pareto front compared to generation 250. This indicates that 250 iterations are sufficient to achieve a stable global optimum, and further increasing the number of iterations does not yield additional benefits. Therefore, to improve optimization efficiency and reduce the convergence time of the PSSAO algorithm, 250 iterations are selected for the optimization of coordinated control and enhancement of URCP surface properties in this study. Moreover, from the distribution of the entire URCP Pareto-optimal solution set, it can be observed that as hardness and residual compressive stress increase, surface roughness also tends to rise. This outcome aligns with the simulation and experimental results presented earlier. The increase in residual stress and the thickening of the strain-hardened layer can lead to increased surface roughness of 42CrMo steel, potentially resulting in surface defects such as micro-cracks and pitting, which may further affect the final surface quality after URCP strengthening. This Pareto front curve also confirms the trade-offs that exist between different surface properties—namely, residual stress, surface roughness, and hardness—in the URCP multi-objective optimization process. Only through the use of the PSSAO algorithm can these conflicting objectives be effectively balanced, thereby enhancing all surface properties simultaneously and determining the optimal process parameter domain. This highlights the necessity of applying multi-objective optimization algorithms to improve URCP surface properties. Based on the optimization performance of the PSSAO algorithm at 250 iterations, the optimal ultrasonic rolling process parameters are determined, providing a valuable reference for further improving the surface properties and machining quality of 42CrMo steel. As shown in Table 6, Table 7, the objective functions and corresponding optimal URCP process parameter domain obtained from the Pareto-optimal front generated by the PSSAO algorithm are presented. In the final section, the optimal parameters derived from the PSSAO algorithm will be used in ultrasonic rolling strengthening experiments on 42CrMo steel to validate the reliability and effectiveness of the optimization results in improving surface properties.

**Table 6:** Pareto optimal solutions obtained by the PSSAO optimization algorithm.

Surface properties	Pareto-optimal solution set
Residual compressive stress	[−1283 MPa, −1296 MPa]
hardness	[60.9 HRC, 61.6 HRC]
Ra	[0.120 μm, 0.156 μm]

**Table 7:** Optimized process parameter domains corresponding to the Pareto front.

Process parameters	Optimal processing parameter range
Static pressure	[520 N, 700 N]
Ultrasonic amplitude	[7 μm, 9 μm]
Workpiece rotational speed	[50 r/min, 180 r/min]
feed rate	[0.05 mm/r, 0.07 mm/r]

## 5 Discussion

**Fig. 28:**
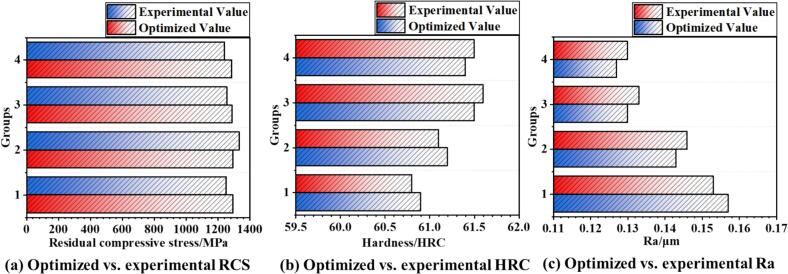
Comparison between the optimized URCP parameter domain and experimental results.

To verify the reliability and rationality of the optimized URCP (Ultrasonic Rolling Composite Process) parameter domain, ultrasonic rolling strengthening experiments were carried out using the optimal processing parameters. Based on the optimal surface performance and corresponding parameter domain obtained in Table 6, Table 7, four sets of Pareto-optimal solutions were randomly selected, along with their corresponding optimal parameter combinations, as shown in Table 8, for URCP tests. Fig. 28 presents the surface performance distributions—surface roughness, residual compressive stress, and hardness—under these four selected optimal processing conditions.

**Table 8:** URCP experimental validation of four randomly selected optimized parameter sets.

Groups	*F*(N)	*A*(μm)	*n*(r/min)	*u*(mm/r)	RCS(MPa)	HRC	Ra(μm)
1	520	9	130	0.05	−1296	60.9	0.157
2	550	8	115	0.07	−1294	61.2	0.143
3	600	8	50	0.05	−1289	61.5	0.130
4	700	7	180	0.06	−1287	61.4	0.127

The results show that the conflicts among different surface performance metrics in URCP were effectively resolved by the PSSAO algorithm. Compared with simulation and experimental data, the surface performance of 42CrMo steel was significantly improved after ultrasonic rolling. The optimized process not only enhanced residual compressive stress and hardness but also improved surface quality. Moreover, a high degree of consistency between the optimization results and experimental data was observed, with all relative errors within 10 %, demonstrating the high accuracy and feasibility of the PSSAO-derived optimal parameter domain.

In contrast, Han et al. [42] found that four rolling passes were required to significantly increase the Vickers hardness of a bronze alloy to 323 HV using ultrasonic rolling. This was largely due to the lack of parameter optimization, which necessitated repeated rolling to improve surface properties. Similarly, Liu et al. [43] needed 10 rolling passes on EA4T axle steel to increase the hardness and residual compressive stress to 340 HV and −850 MPa, respectively. Although ultrasonic rolling enhances surface quality and induces beneficial residual stress, excessive passes reduce process efficiency, contradicting the goals of high-efficiency and green manufacturing. Therefore, using intelligently optimized URCP parameters to achieve enhanced surface performance in a single pass is both effective and essential, aligning with sustainable and energy-efficient manufacturing practices. This significantly improves processing efficiency and reduces energy consumption and carbon emissions in actual production.

**Fig. 29:**
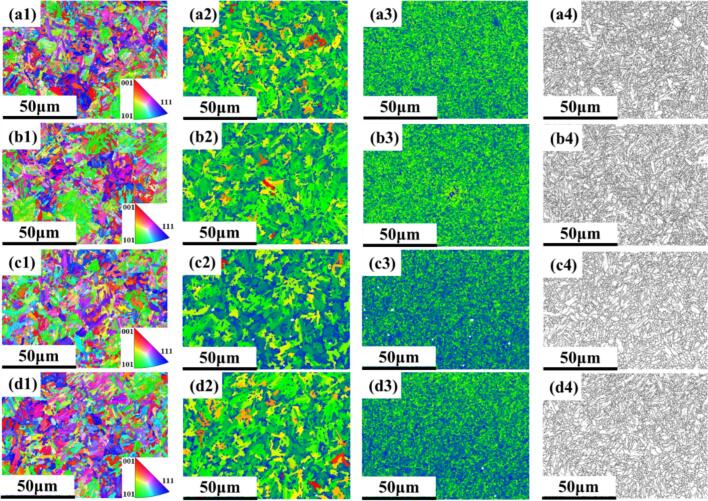
EBSD analysis of the optimized URCP parameter domain. (a1)–(a4): IPF, GOS, KAM, and grain boundary maps for the first set of optimized URCP parameters; (b1)–(b4): IPF, GOS, KAM, and grain boundary maps for the second set of Pareto optimal solutions; (c1)–(c4): IPF, GOS, KAM, and grain boundary maps for the third set of optimal URCP parameters; (d1)–(d4): IPF, GOS, KAM, and grain boundary maps for the fourth set of optimized process parameters.

Furthermore, to verify the microstructural strengthening mechanisms underlying the improved surface properties under the optimized URCP parameters (Table 8), EBSD analysis was conducted on 42CrMo steel using a Crossbeam 350 SEM equipped with an Oxford detector. To ensure high-quality Kikuchi patterns and improved spatial resolution during EBSD analysis, the specimens were subjected to final surface preparation using an argon ion milling system (EOL-19530CP). The ion polishing was conducted at an accelerating voltage of 7.5 kV for 30 min, with an argon ion current of 11.3 μA, which provided a deformation-free surface suitable for EBSD examination. The EBSD scans were taken approximately 100 μm below the surface. Fig. 29a–d correspond to EBSD maps for the four selected optimal Pareto solutions. Fig. 29a1–29d1 show that the grains on the 42CrMo surface after URCP mainly exhibit < 001 > and < 101 > orientations. Fig. 29a2–d2 illustrate the Grain Orientation Spread (GOS) distribution for each group, representing the average angular deviation within individual grains. The measured GOS values were 8.69°, 9.28°, 10.47°, and 8.59°, respectively. These increased GOS values indicate severe plastic deformation induced by ultrasonic rolling, which elevates the residual compressive stress and dislocation density in the surface layers. As shown in Fig. 28, the formation of a residual stress layer and increased hardness on the material surface is mirrored microscopically by the elevated GOS. The rise in GOS also indicates the formation of a high-density gradient structure near the surface, which synergistically contributes to the improved surface properties. Fig. 29a3–d3 show the Kernel Average Misorientation (KAM) distributions. KAM values increased to 5.0, 4.99, 4.95, and 4.74, respectively, under the optimized parameters. These elevated KAM values confirm that URCP causes severe lattice distortion and promotes dislocation strengthening and plastic strain, which are consistent with the elevated GOS values. Together, they reflect the increased internal dislocation density and localized strain, resulting in material hardening and improved hardness. Fig. 29a4–d4 display the grain boundary characteristics under the optimized processing conditions. A significant increase in total grain boundary count is observed, especially in the fraction of low-angle grain boundaries (LAGBs), which reached 71 %, 70.6 %, 69.8 %, and 70.2 %, respectively, while high-angle boundaries dropped below 30 %. The rise in LAGBs is associated with grain refinement and dislocation multiplication. Furthermore, the shift in grain orientations toward 〈0 0 1〉 and 〈1 0 1〉 promotes sub-grain formation and structural evolution, intensifying the LAGB proportion. These microstructural changes elevate the dislocation density and promote material hardening, thereby enhancing the surface performance of 42CrMo steel. For comparison, Zou et al. [44] achieved a 70 % LAGB ratio and a KAM value of only 1.5 in 7075 aluminum alloy after three ultrasonic rolling passes, with surface roughness reduced to 0.256 μm. In contrast, the present study achieved superior surface performance improvements in 42CrMo steel with just one rolling pass. This clearly demonstrates the effectiveness and necessity of the proposed PSSAO multi-objective optimization algorithm. It plays a critical role in enhancing surface properties and promoting efficient, low-emission manufacturing.

In conclusion, applying the newly proposed PSSAO algorithm to optimize URCP parameters significantly enhances the surface performance and quality of 42CrMo steel. On a microstructural level, grain refinement, increased LAGB density, and elevated dislocation density contribute to improved mechanical properties and surface integrity, thereby enhancing fatigue resistance and service life. These improvements, achieved in a single rolling pass, embody the synergistic enhancement of efficiency and low energy consumption. This aligns with the goals of sustainable manufacturing and carbon reduction, and highlights the promising potential of ultrasonic rolling in green manufacturing applications.

## 6 Conclusions

This study investigates the surface performance enhancement and optimal parameter domain for URCP of 42CrMo steel through theoretical analysis, numerical simulation, machining experiments, and multi-objective optimization. The results demonstrate the feasibility of efficient processing and surface enhancement of 42CrMo steel rods using URCP, contributing to green manufacturing with reduced energy consumption and carbon emissions. Compared with conventional rolling, ultrasonic rolling significantly improves the surface properties and processing efficiency of 42CrMo steel, achieving both high-efficiency machining and the objectives of sustainable manufacturing. The main conclusions of this study are as follows:

(1) The transducer in the ultrasonic rolling device converts the electrical signals generated by the ultrasonic generator into mechanical vibrations. Through resonance between the external excitation frequency and the device's natural frequency, and amplified by the stepped horn structure, high-frequency ultrasonic energy is transmitted via the rolling ball onto the surface of 42CrMo steel. Compared with traditional rolling and grinding, ultrasonic rolling introduces beneficial residual compressive stress and work hardening layers on the material surface, enhancing mechanical properties while reducing surface defects. Moreover, the process avoids structural damage and coolant consumption, thereby achieving a synergistic effect between energy-efficient manufacturing and reduced emissions.
(2) The established thermomechanical coupling model accurately simulates the interaction between the stress and temperature fields during ultrasonic rolling. The localized temperature rise generated during URCP softens the material in the processing zone, promoting intense plastic deformation and enhancing the formation of the hardened surface layer, while slightly compromising surface roughness. It was also found that surface mechanical properties such as residual compressive stress and hardness increase with higher static pressure and ultrasonic amplitude but decrease with increasing spindle speed and feed rate. In contrast, surface roughness decreases with increasing amplitude and static pressure, but shows a trend of first decreasing and then increasing with rising spindle speed and feed rate.
(3) A novel PSSAO algorithm was proposed to effectively resolve the conflicting objectives in optimizing the surface properties of 42CrMo steel after URCP. The URCP predictive model, constructed via response surface methodology, was coupled with the PSSAO algorithm to perform multi-objective optimization. The results show significant improvements in surface performance and surface quality. The optimal processing parameter ranges were determined as follows: feed rate from 0.05 mm/r to 0.07 mm/r, static pressure from 520 N to 700 N, ultrasonic amplitude from 7 μm to 9 μm, and spindle speed from 50 r/min to 180 r/min. Under these optimized conditions, the residual compressive stress reached −1283 MPa to −1296 MPa, Ra was reduced to 0.120 μm to 0.156 μm, and hardness was improved to 60.9 HRC to 61.6 HRC. These findings validate the high efficiency and eco-friendly potential of ultrasonic rolling, contributing to reduced energy consumption and carbon emissions.

## CRediT authorship contribution statement

**Haojie Wang:** Writing – original draft, Methodology, Investigation, Formal analysis, Data curation, Conceptualization. **Xiaoqiang Wang:** Software, Resources, Project administration, Funding acquisition, Supervision. **Eric Velázquez-Corral:** Supervision, Software, Resources. **Ramón Jerez-Mesa:** Writing – review & editing, Visualization, Validation, Supervision, Project administration.

## Declaration of competing interest

The authors declare that they have no known competing financial interests or personal relationships that could have appeared to influence the work reported in this paper.
